# Impact of side hole design of drainage cannulas on flow velocity and predilection sites for low-flow areas in veno-venous ECMO circuits

**DOI:** 10.1186/s40635-026-00980-8

**Published:** 2026-10-09

**Authors:** Markus Bongert, Vincent Marciniak, Wolfram Windisch, Christian Karagiannidis

**Affiliations:** 1 ARDS and ECMO Centre Cologne-Merheim, University Witten/Herdecke, Alfred-Herrhausen-Straße 50, 58455 Witten, Germany; 2 Department of Mechanical Engineering, University of Applied Sciences and Arts Dortmund, Sonnenstr. 96, 44139 Dortmund, Germany; 3 Center of Research in Biomedical Engineering, University of Applied Sciences and Arts Dortmund, Sonnenstr. 96, 44139 Dortmund, Germany; 4 Department for Human Medicine, University Witten/Herdecke, Dipl.-Ing. Markus Bongert, Alfred-Herrhausen-Straße 50, 58455 Witten, Germany

**Keywords:** Drainage cannula, Side hole, Cannula design, Computational Flow Dynamic (CFD), Flow pattern, ECCO2R

## Abstract

**Background:**

Veno-Venous Extracorporeal Membrane Oxygenation (VV-ECMO) (VV-ECMO) and Veno-Artial Extracorporeal Membrane Oxygenation (VA-ECMO) have become evidence-based therapy options for patients with most severe heart and/or lung failure. Cannulas continue to play a central role as the direct interface between patients and extracorporeal circulation. However, severe side-effects, such as coagulation and haemolysis, may result not only from a blood pump but also from inappropriate cannula design.

**Methods:**

Based on the multi-stage lighthouse design of a draining ECMO cannula, the area of the side holes was varied in both shape and size in the distal and proximal directions. Using a Computer-Aided Design (CAD)- Model of cannulas (19,21,23 French (Fr)) appropriate simulation models were created in the non-reactive plenum. The resultant numerical 3D simulations are based on physiological boundary conditions and on the flows covering different ECMO-flow rates.

**Results:**

The current cannula design reveals no-flow zones, which induce thrombus formation, especially when using blood-flow rates below 2 L/min. New geometric cannula design variants revealed an improvement in pressure loss, but at the expense of pronounced no-flow zones in the distal region. During drainage, the largest proportion of the total volume flow drains through the proximal row of side holes. Here, the pressure and thus the flow for both, immediately drops to the level of the surrounding plenum. This means that risk areas for haemostasis develop more distally. The direct influence of the design is evident in the proximally exponentially reduced side holes. They significantly reduce the extent of the no-flow zones.

Simulations of the reference cannula showed that suction to the vessel wall and thus closure of some side holes contributes to an overall improvement in flow within the cannula.

**Conclusions:**

When reducing the blood-flow rate in ECMO therapy, stagnation areas may develop within the drainage cannula. The geometry of the side holes has an important impact. A comparison of the different designs shows that distal growth of the side hole area results in improved flow behaviour.

**Supplementary Information:**

The online version contains supplementary material available at https://doi.org/10.1186/s40635-026-00980-8.

## Background

Veno-Venous Extra Corporeal Membrane Oxygenation (VV-ECMO) is an evidence-based therapy for patients suffering from severe acute respiratory failure [1, 2]. In cases of severe heart failure, Veno-Artial Extracorporeal Membrane Oxygenation (VA-ECMO) acts as an artificial heart and lung [3]. The same cannulas have been used for both techniques for decades now. Side effects such as clotting may not only occur from oxygenators and blood-pumps, but also and underestimated from cannulas. New cannula design has focused on replacing double single lumen cannulas with a single, double lumen cannula [4], but much less on optimazation of the flow and especially reduction of stagnation areas, which occur in every cannula, even more pronounced in lower flow ranges. Current research focuses around self-expanding cannulas [4–7] This study focused on thin-walled cannulas, which are primarily used in every day clinical practice.

Although ECMO is often life-saving, it may cause severe complications in up to 10% of cases [8–10]. Some of these complications stem from bleeding and clotting caused by the foreign surface or an Acquired van-Willebrand Syndrom (AvWS) due to the blood pump. Therefore, systemic anticoagulation is a key to reduce clotting comlications. However it is usually impossible to fully reverse the effects of the artificial surface. Therefore, clotting complications usually do occur less in the beginning of the treatment and mainly after 7–10 days. It has been shown [11] that at flow rates below 2 L/min, complications such as haemolysis and thrombus formation occur with an increased risk of bleeding. However, in recent years, less attention has been paid to the role of cannulas as a major cause of complications. There are areas within the cannula, where hardly any blood flow is evident. Stagnation, which occurs regularly in cannulas may be one reason why clotting occurs.

Particular attention must be paid to drainage. Whereas the tip of the drainage cannula (distal) should ensure adequate blood flow, it should not have an adverse effect on the blood. This means that not only the diameter, which is limited by the dimensions of the venous vessel, but also the design of the cannula is of particular importance. In addition to the fluid mechanics fundamentals of ECMO cannulas, Kohler's work [12] covers key aspects of ideal cannula design and its impact on blood flow. To date, pressure loss curves have been used to describe the performance of cannulas. This parameter is of importance both to minimise suction pressure in drainage cannulas, and yet maximise volume flow to avoid haemolysis. However, the flow itself and the velocity distribution within the cannula is equally important in determining performance. Almost all cannulas available on the market have side holes designed to ensure full drainage even when the tip of the cannula is closed. Cannula design differs mainly in the number and position of side holes, the geometric shape of which is also an important design feature. Conventionally, viewed in longitudinal section, the walls of the side holes are perpendicular to the longitudinal axis of the cannula. Park has shown the influence of side hole number of wall angle on venous cannula performance [13]. Using Computational Fluid Dynamics (CFD), an increase in flow and a reduction in wall shear stress (WSS) were determined. In the study by Vatani et al. [14], sequential variation of key geometric design parameters (diameter, spacing, angle) of the side holes significantly reduced WSS and increased the speed at the cannula exit, thereby minimising the risk of thrombosis. The results [15] confirm the influence of the number and size of the side holes at the tip of a drainage cannula on the likelihood of thrombosis formation, along with improved overall performance. Different groups [16–18] have independently observed that in both multi-stage and single-stage cannulas with a lighthouse tip flow is greatest through the most proximally located side holes and decreases rapidly towards the tip. One key finding [19] is the critical number of side holes. However, increasing the number of side holes does not result in any additional improvement in drainage efficiency. The in silico study by Takeshi et al. [20] suggests that energy loss occurs due to flow separation at the side holes and volume flow is inversely proportional to the number of side holes for the same total opening area. Rorro [21] has shown that a non-centred position of the cannula had no substantial influence on the results of the research groups mentioned above.

We therefore determined the current design of the draining cannulas with their drawbacks and proved different new design variants, which may overcome the current problems.

## Methods

### Aim

Since the contribution of cannulas to ECMO complications, especially clotting, has been underestimated to date, the aim of this study was to identify areas of stagnation within standard cannulas of ECMO therapy, which may promote clotting due to stagnation. Furthermore, the study aims to find an optimized design of the side holes in a drainage cannula, targeting the reduction of high-risk internal stagnation areas and, secondarily, the pressure loss behaviour.

### Study design

**Fig. 1:**
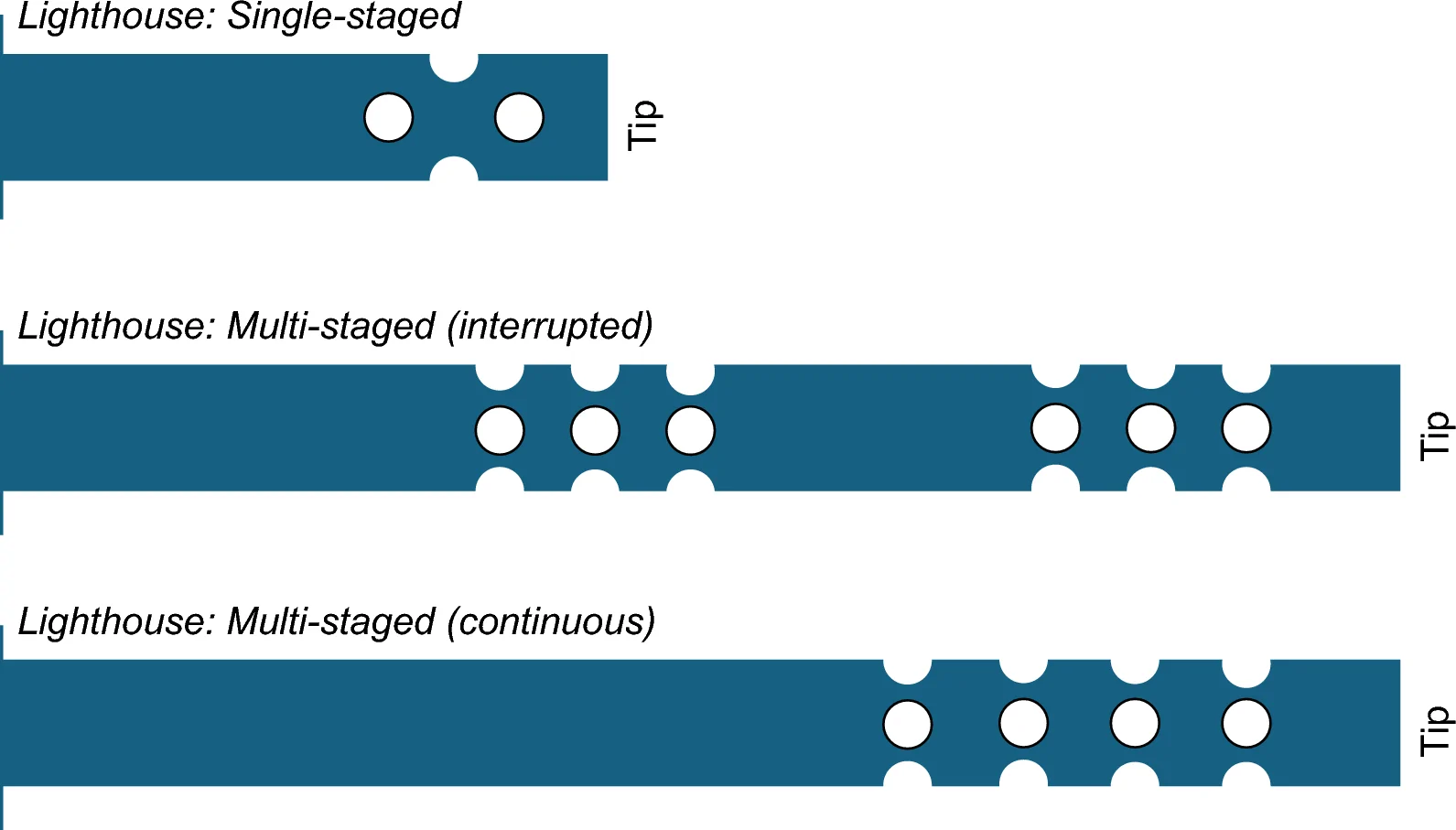
Visualisation of the most common cannula lighthouse designs. Cannulas can be classified into three basic lighthouse designs. They differ in the positioning of the side hole rows and in the number of side holes per row. (Top) Single-staged, (Middle) Multi-staged interrupted, (Bottom) Multi-staged continuous

A large number of different cannulas designs are available for ECMO therapy [22]. Cannulas can be classified into three basic lighthouse designs: Single-staged (side holes only at the tip area), Multi-staged-interrupted and Multi-staged-continuous (Fig. 1). In the single-staged design, the side holes are located only in the head area of the cannula, with two always opposite each other and offset by 90°. The multi-staged interrupted design features several packages positioned at equal intervals, consisting of one or more rows of side holes with four openings opposite each other. In contrast, in the multi-staged continuous variant, the rows of side holes with their four openings are distributed continuously at equal intervals (Table 1).

**Table 1:** Summary of abbreviation and their meaning

Basic design	Position of rows	Side hole shape	Abbreviation
Lighthouse	Multi-staged, Continuous	Circle	MSC-LH-CI
Lighthouse	Multi-staged, Continuous	Elliptical	MSC-LH-EL
Lighthouse	Multi-staged, Continuous	Slothole	MSC-LH-SL
Lighthouse	Multi-staged, Continuous	Slothole proximal	MSC-LH-SP
Lighthouse	Multi-staged, Continuous	Slothole distal	MSC-LH-SD
Lighthouse	Multi-staged, Continuous	Circle (2 lines closed)	MSC-LH-CI-2Q
Lighthouse	Multi-staged, Continuous	Circle (3 lines closed)	MSC-LH-CI-3Q
Lighthouse	Single-staged	Circle	SS-LH-CI

In this study, based on the design MSC-LH-CI of a Maquet® venous cannula (Getinge® AB, Lindholmspiren 7, P.O. Box 8861, SE−417 56, Gothenburg, Sweden), the side holes were investigated to determine the extent to which the variation of the cross-sectional area or geometric design influences pressure loss and flow behaviour within the cannula.

Starting from the circular area of the side holes of the reference cannula (MSC-LH-CI), its shape has been enlarged by varying it in the longitudinal direction. To do this, the circular area has been halved and pulled apart, creating a slot hole (MSC-LH-SL). The next variant features elliptical side holes (MSC-LH-EL) instead of slotted side holes.After varying only one parameter (surface shape), the surface area was changed as an additional parameter for the other two design variants. This means that, on the one hand, the side holes have been enlarged proximally from a circle to an slot hole (MSC-LH-SP). On the other hand, the side holes have been enlarged distally from a circle to an slot hole (MSC-LH-SD).

Furthermore, the influence side hole position was investigated using the single-staged lighthouse (SS-LH-CI) design. Due to the single-staged lighthouse design (SS-LH-CI), all side holes are clustered together and not arranged in rows. Therefore, the term ‘Head’ is used for the region of interest instead of ‘ROW x’. Finally, the situation was evaluated for the reference design (MSC-LH-CI) when two or even three rows of side holes are occluded, e.g., by suction to the vessel wall.

All investigations were carried out using CFD. Through its accuracy, the use of CFD has proven itself in many areas of medical technology [23–26]. In addition to comparing both the numerical and graphical results, a statistical analysis is also carried out.

### Modeling

**Fig. 2:**
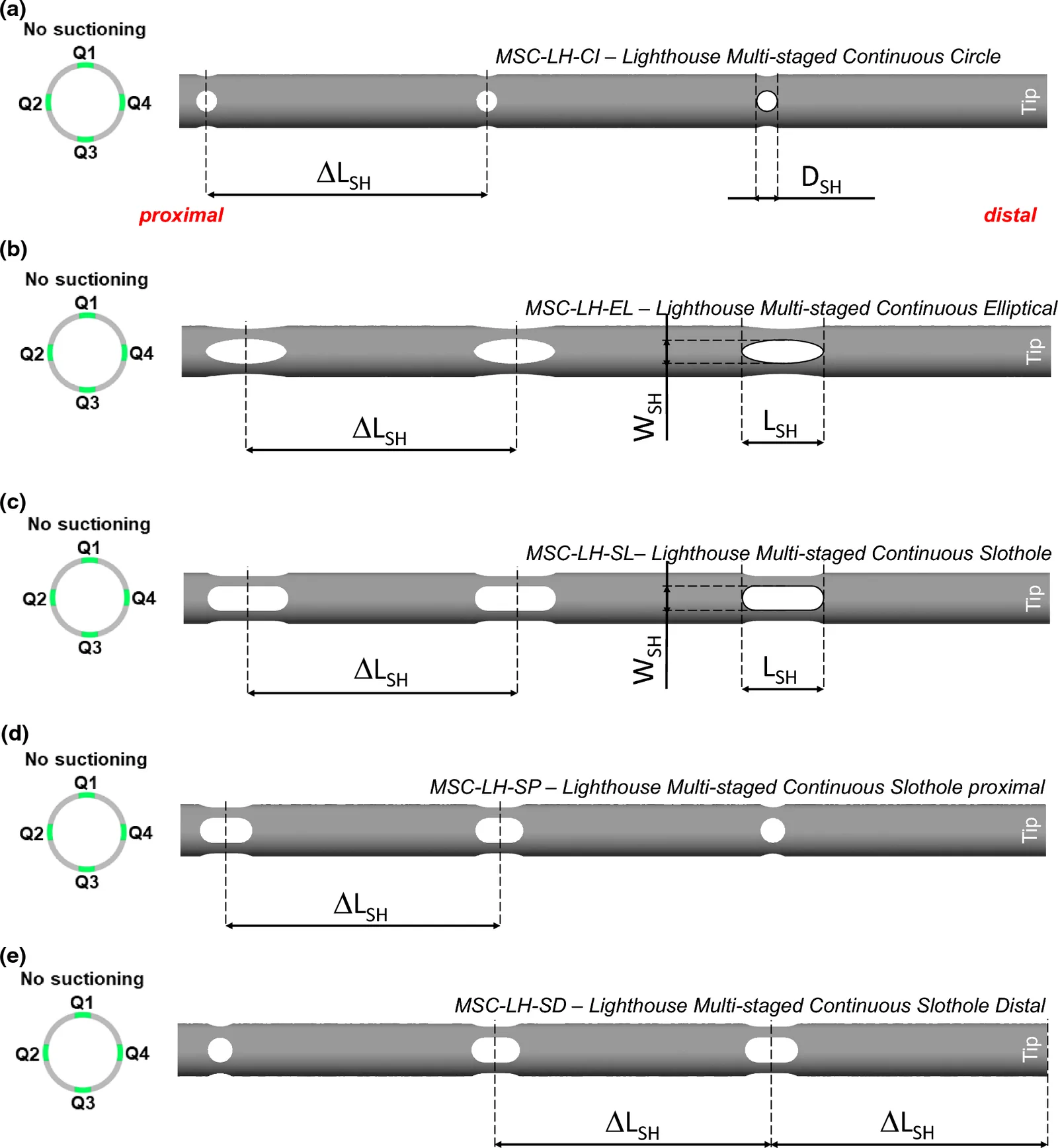
Illustration of the side hole designs analysed in this study. (**A**) Circular, (**B**) Elliptical, (**C**) Slothole, (**D**) Slothole proximal and (**E**) Slothole distal. In all designs, two side holes Q_i=1,4_ have been arranged opposite each other. The green marking has been chosen as a symbol for an unclosed side hole Legend. side hole (ΔL_SH_), Diameter side hole (D_SH_), Width side hole (W_SH_)

**Fig. 3:**
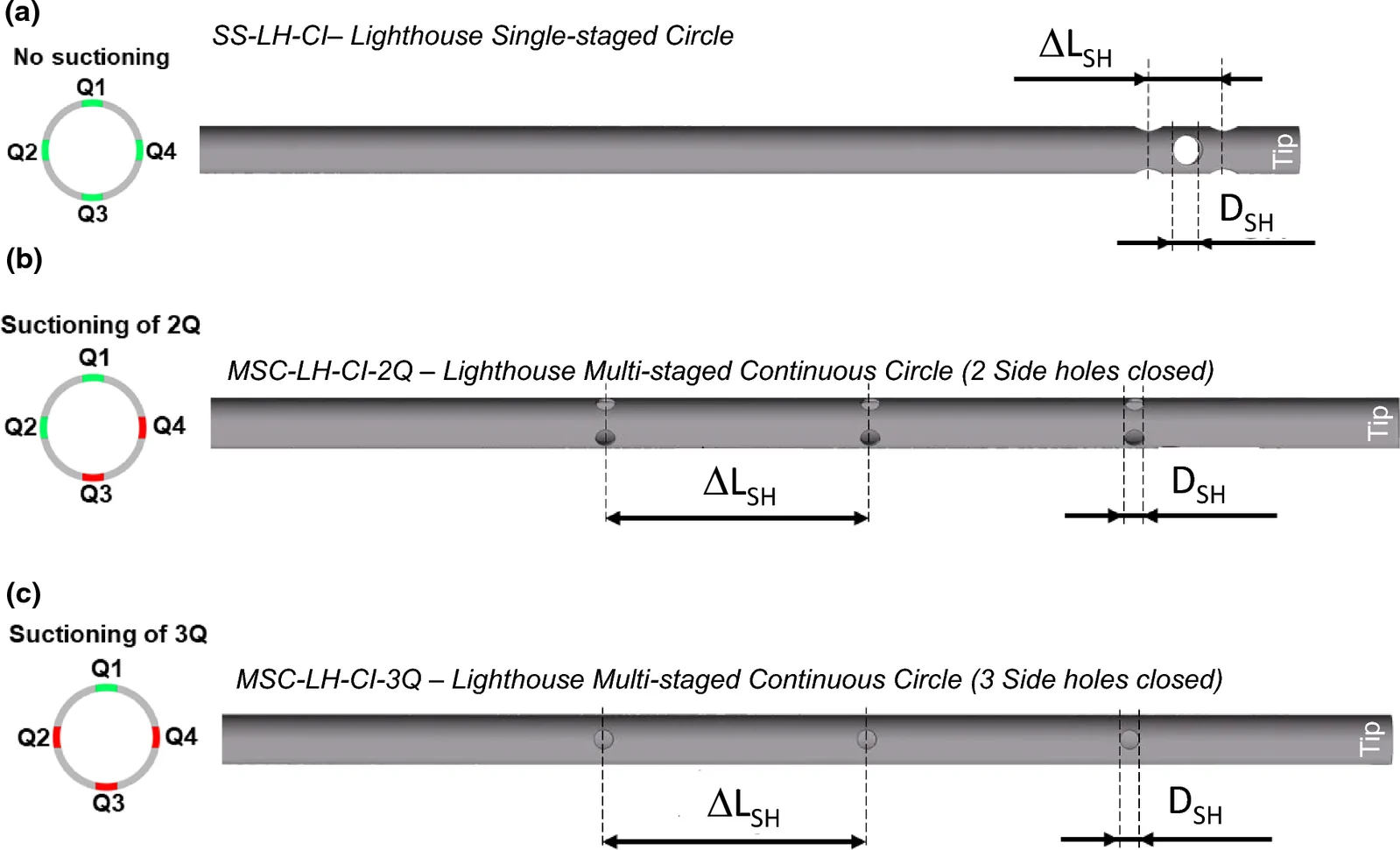
Illustration of single-staged design and suctioned side hole rows analysed in this study. (**A**) Lighthouse, (**B**) Multi-staged 2 Side holes closed, (**C**) Multi-staged 3 Side holes closed. In LH, two side holes Q_i=1,4_ have been arranged opposite each other. The red marking has been chosen as a symbol for a closed side hole. Legend: Length side hole (ΔL_SH_), Diameter side hole (D_SH_), Width side hole (W_SH_)

The 3D CAD models of the cannulas with the three most common outer diameters of 19 Fr., 21 Fr. and 23 Fr. were created using SpaceClaim® from Ansys® (Southpointe 2600 Ansys Drive, Canonsburg, PA 15317 USA). Further details on the structure and design are visualised in Figs. 2 and 3. The selected diameters (19 Fr., 21 Fr. and 23 Fr.), flows (1.0 – 6.0 l/min) and cannula designs are standard in the clinical setting at the ARDS and ECMO Centre Cologne-Merheim and were therefore used for this study.

### 3D Flow simulation

Based on the 3D CAD models of the variants analysed, models of the fluid volumes were generated, which in turn, together with the physiological boundary conditions used, formed the basis for the flow simulations. These flow simulations were carried out with the CFD solver Fluent® (Ansys®). The results of all flow simulations were analysed both numerically and visually, and compared with each other. Pressure loss (Supplement A), drainage performance and flow behaviour, including flow direction, were defined as evaluation parameters.

A more detailed description of the structure of the simulation models and the boundary conditions for the simulations can be found in Supplement B.

### Statistical analysis

A one-way ANOVA test was used for the statistical analysis. This one-factor variance analysis makes it possible to examine whether different changes/levels have a measurable effect on a dependent variable when there is a single independent factor. The position of the side holes between the cannula tip and all side hole rows was selected as the independent factor. The standardised core velocity v/v_max_ along the central axis of the cannula was used as the dependent variable. A significance value (p-value) below 0.05 was considered statistically significant.

During post-processing, the core velocity along the central axis was read out at the centre of each side hole. Standardisation was then carried out to improve comparability between the cannulas. The Prism 10 software from GraphPad (Dotmatics, 225 Franklin Avenue, 26th Floor, Boston, Massachusetts, 02110, USA) was used to perform the one-way ANOVA test.

## Results

### Drainage performance

The flow behaviour of the side hole rows is described below using the reference cannula as an example (Table 2). The behaviour is similar in principle for all other design variants. Only the numerical values vary considerably in some cases. All data for all cannulas are listed in Supplementary Tables 4–11 in Supplement A.

**Table 2:** Reference design (MSC-LH-CI): Operating data and drainage performance

				Flow				
Cannula	Operating flow [L/min]	Pressure loss [mmHg]	Max. velocity [m/s]	(Row IV) [%]	(Row III) [%]	(Row II) [%]	(Row I) [%]	(Tip) [%]
MSC-LH-CI-19	1,0	-14,1	1,1		61,1	27,2	10,7	1,0
	2,0	-38,4	2,1		58,0	26,5	11,2	4,3
	2,5	-55,2	2,6		57,1	26,4	11,3	5,6
	3,0	-74,4	3,1		56,4	26,3	11,4	5,8
	4,0	-119,5	4,1		55,5	26,2	11,5	6,8
	5,0	-173,5	5,0		54,8	26,1	11,6	7,6
	6,0	-236,4	6,0		54,2	26,0	11,7	8,1
MSC-LH-CI-21	1,0	-9,3	0,9		53,2	27,0	12,4	7,5
	2,0	-24,2	1,7		50,5	26,3	12,7	10,5
	2,5	-34,5	2,1		49,7	26,1	12,8	11,4
	3,0	-46,7	2,5		49,2	26,0	12,8	12,0
	4,0	-75,3	3,2		48,9	26,2	13,1	11,8
	5,0	-109,3	4,0		47,6	25,7	13,0	13,7
	6,0	-148,7	4,8		47,1	25,8	13,1	14,2
MSC-LH-CI-23	1,0	-6,4	0,7	45,6	26,0	14,5	7,1	6,8
	2,0	-16,4	1,4	43,5	25,4	14,5	7,6	9,0
	2,5	-23,0	1,7	42,9	25,3	14,5	7,7	9,6
	3,0	-30,8	2,1	42,8	25,2	14,6	7,8	10,1
	4,0	-50,0	2,7	41,7	25,0	14,6	8,0	10,8
	5,0	-72,9	3,4	41,1	24,8	14,7	8,1	11,4
	6,0	-99,2	4,0	40,7	24,7	14,7	8,2	11,8

For the reference design (MSC-LH-CI), the percentage shares of the side holes in the total extracted lumen are summarised for all selected diameters and relevant volume flows. The proximal row of side holes (Row III or Row IV) extracts more than half of the total volume. The percentage is reduced further distally.

Maximum velocity’ in a 19 Fr. (21 Fr., 23 Fr.) cannula is almost directly correlated to flow in ECMO, such that at a flow rate of 1.0 L/min, a maximum velocity of 1.1 m/s (0.9 m/s, 0.74 m/s) is observed, and at a rate of 5.0 L/min, a velocity of 5.04 m/s (3.98 m/s, 3.35 m/s). This relationship changes with the diameter of the catheter. As it increases, the velocity decreases.

Suction pressure increases significantly with increasing ECMO flow. With the 19 Fr. variant, a negative pressure of − 173.49 mmHg is needed to produce an ECMO flow rate of 5.0 L/min, with traumatic consequences for blood integrity, such as haemolysis [27, 28]. The existence of a jump range with regard to suction pressure could be determined, i.e. between two ECMO flows, the suction pressure increases by approximately 30 to 60 mmHg. With the 19Fr. and 21 Fr. cannulas, this range begins at 3.0 L/min. No such jumps can be detected for the 23 Fr. diameter.

The percentage proportions for the other distal side hole rows change only slightly as the ECMO flow increases.

When looking at the percentages of the side holes (Table 2), it can be seen that the proximal row of side holes of the reference design MSC-LH-CI with 19 Fr. with an average value across all volume flows of 56.7% extracts the largest proportion from the lumen surrounding it. In the distal direction, this percentage is always reduced by around half compared to the previous row. For the 21 Fr. and 23 Fr. diameters, the middle, distal, percentage share of the drained volume is reduced to 49.5% and 42.5% respectively in favour of the proximally placed rows of side holes. However, the last proximal row of side holes and the tip of the cannula are excluded from this. Their values hardly differ in terms of amount (1.2% and 2.1% respectively).

**Fig. 4:**
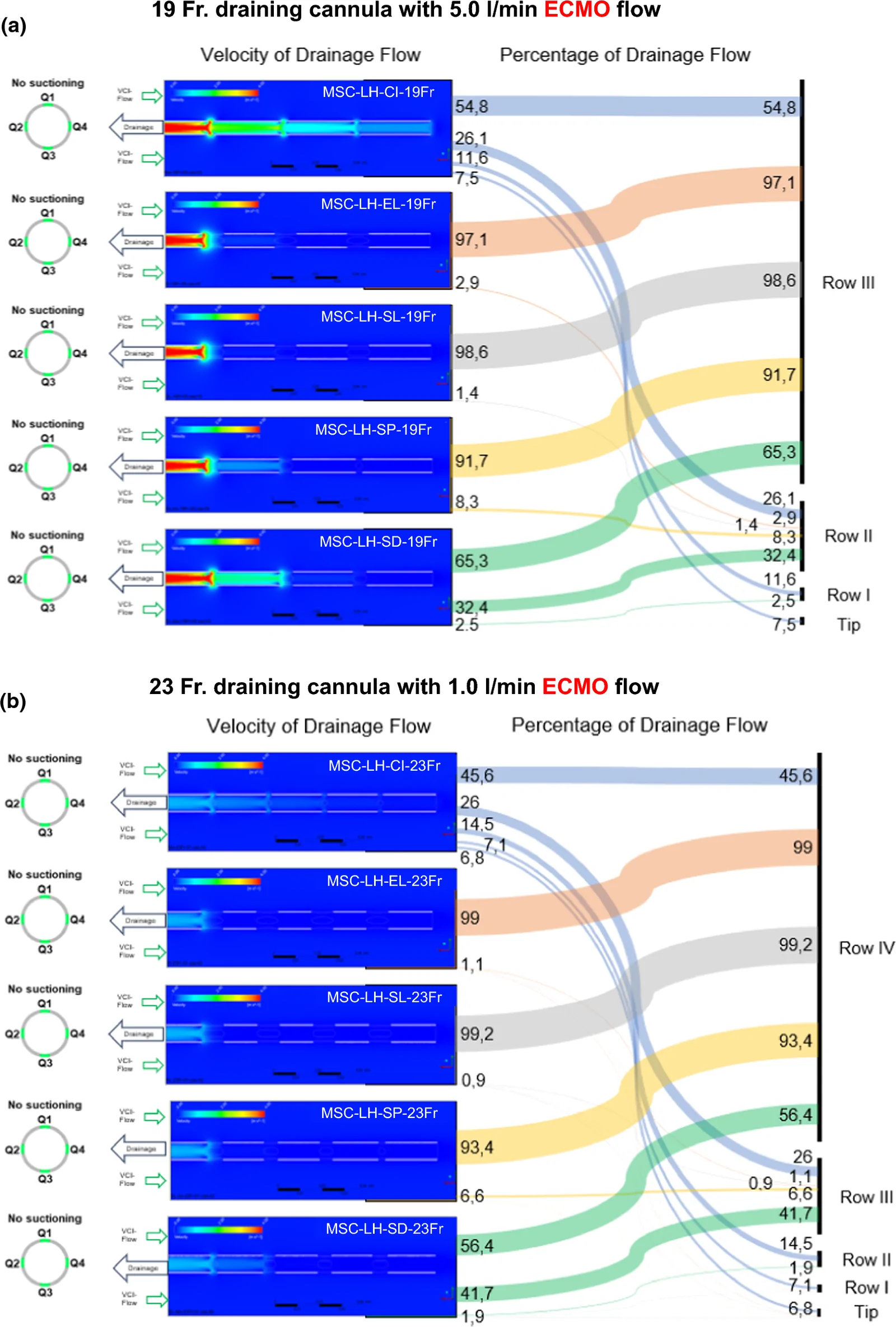
Cannula drainage performance for different boundary conditions. The velocity distribution within the cannulas shown in the middle graphs clearly visualises from which row of side holes the most is drained. The qualitatively visualised distribution of the drained volume flow across the rows of side holes is shown quantitatively in the graph on the right. Only the reference design (MSC-LH-CI) manages to drain a significant proportion of the total volume through the cannula tip. (**A**) Combination of 19 Fr and 5.0 L/min, (**B**) Combination of 23 Fr and 1.0 L/min, Velocity range: 0.0 – 4.0 l/min

Figure 4A and B visualises the percentage distribution of drained fluid volume for individual side hole rows for the respective variants of the cannula design. For this purpose, these two combinations of ECMO flow and diameter have been selected in which the flow situations with the highest (A) and lowest (B) flow velocity within the cannula are generated. All other combinations are in intermediate ranges. Therefore, it is sufficient to consider only the two extremes when assessing the influence of the side holes in the further course of the analysis. Full suction is possible in all four quadrants. The reference design (MSC-LH-CI) has a large area with a safe flow velocity (greater than 0.1 m/s). However, only 7.5% of the ECMO flow passes through the tip of the cannula. The variants MSC-LH-EL, MSC-LH-SL, MSC-LH-SP have an almost identical velocity distribution. Looking at the flow proportions in the proximal side hole row, there is also only a slight difference between MSC-LH-EL and MSC-LH-SL (approx. 99%) and MSC-LH-SP (approx. 93%). The MSC-LH-SD design, as the best variant with an enlarged cross-sectional area of the side holes, also has further potential for optimisation. This means the largest possible area with a high and homogeneous velocity, including a flow fraction of at least 7.5% through the cannula tip. The same findings apply to the 23 Fr & 1 L/min combination as to the 19 Fr & 5 L/min combination. However, the percentage shares per side hole row are lower. Velocity ranged from 0 to 4 m/s for all variants.

### Flow behaviour

**Fig. 5:**
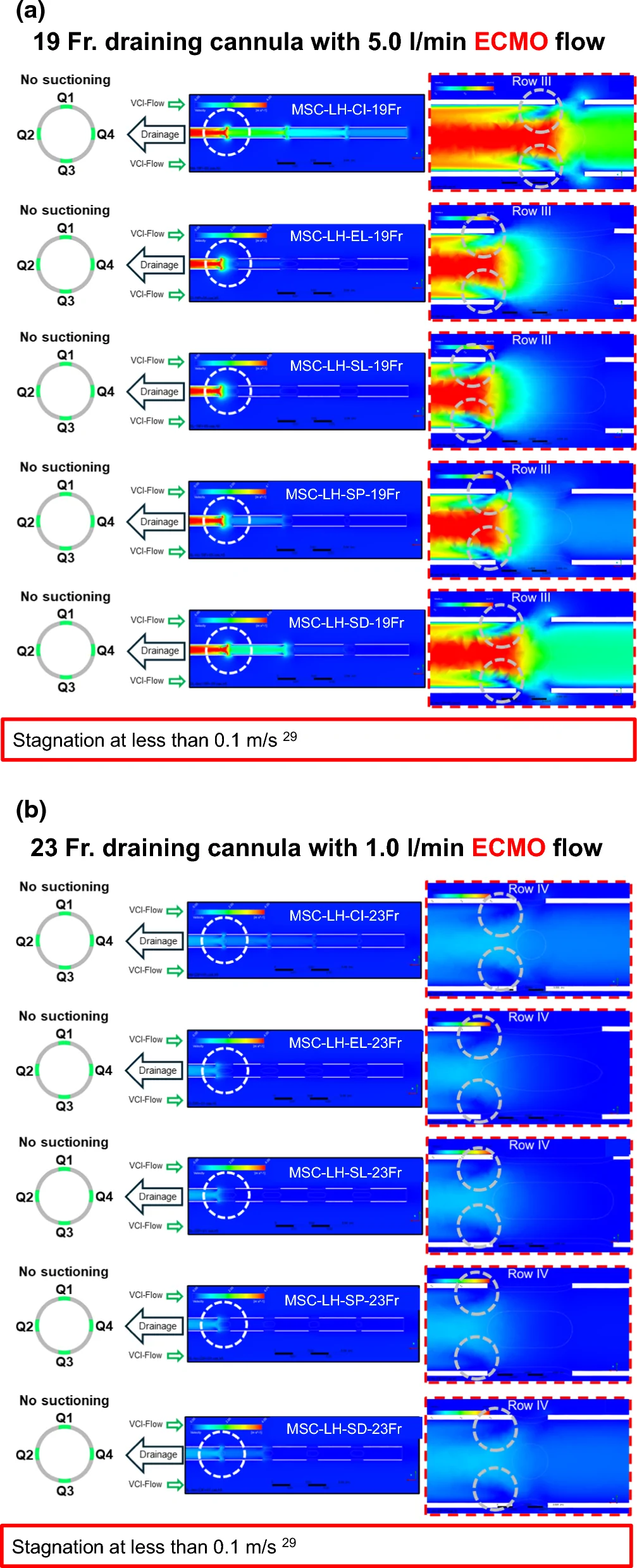
Velocity distribution within cannula for different side holes designs. The side holes in all four quadrants are free. For all design variants, the greatest flow velocity and therefore also volume flow exists at the first proximal row of side holes. Distally, the flow velocity is reduced and the risk of stasis increases. This applies in particular to the combination of a 23 Fr. cannula and an ECMO flow of only 1.0 L/min. The detailed view of this row of side holes (dashed circle) shows the formation of symmetrically distributed funnel-shaped zones with flow velocities of almost 0.0 m/s (dashed circles in the right-hand diagram). (**A**) Combination of 19 Fr and 5.0 L/min, (**B**) Combination of 23 Fr and 1.0 L/min, Velocity range: 0.0 – 4.0 l/min

The velocity distribution in Fig. 5 was used to assess flow. For this purpose, it was visualised on the central cross-sectional area (2D) inside and in the immediate surrounding area outside the respective design along the cannula. The range from 0.0 to 4.0 m/s was selected for the scaling to visualise low velocities zones. The side holes in all four quadrants were free. From the possible combinations of diameter and ECMO flow, the two variants that produced an extreme flow situation velocity distribution were selected.

The maximum possible velocity is achieved with an ECMO flow of 5.0 L/min for the cannula diameter of 19 Fr. Figure 5A compares the speed distribution of the analysed design variants of the side holes. The reference design (MSC-LH-CI), with the circular side holes, showed velocities of approx. 1.0 m/s even in the most distal row of side holes (Row I). Even at the tip of the cannula, velocities of approx. 0.5 m/s still occured. The observation of the velocity distribution for the two variants MSC-LH-EL and MSC-LH-SL visually confirms the extreme suction behaviour of these design variants, already apparent in the different percentages of the side hole rows in the suctioned volume. Maximum velocities of at least 1.0 m/s were only achieved in the proximal row of side holes (Row III). However, the velocity droped rapidly to below 0.5 m/s from the centre of the side hole. In the distal rows of side holes (Row II and Row I) and at the tip of the cannula, the speed dropped further to approx. 0.0 m/s. It should be noted that below 0.1 m/s, stasis, i.e. clotting, develops [29] (Supplement C).

The exponential, distal reduction in the surface area of the side holes resulted in only a marginal improvement in flow for the Row III. However, velocities of approx. 0.5 m/s were achieved between Row III and Row II and up to half of Row II. The velocity only dropped to 0.0 m/s in the further course. The exponential, proximal reduction in the area of the side holes, on the other hand, normalises the speed in Row III, i.e. it is at least 1.0 m/s for the entire diameter of the side hole. It also increased to at least 1.0 m/s in the further course up to half of Row II. Even in the area up to Row I, a speed of approx. 0.5 m/s occurred. Only then did it drop to 0.0 m/s up to the tip of the cannula.

The lowest possible speed was achieved with an ECMO flow of 1.0 L/min for the cannula diameter of 23 Fr. Figure 5B also shows the respective distribution of the velocity for this combination for the different design variants of the side holes. If these velocities are compared with those for an ECMO flow of 5.0 L/min, they have an almost identical distribution. Only the level is correspondingly lower. It is noteworthy that even the reference design (MSC-LH-CI) has a velocity of only 0.1 m/s in the area between Row I and Tip. In the MSC-LH-EL, MSC-LH-SL and MSC-LH-SP variants, the range increased with a speed of 0.0 m/s from the tip of the cannula to Row IV. Only the MSC-LH-SD variant can exhibit a velocity of at least 0.5 m/s, at least in the two proximal side hole rows (Row IV and Row III). Then, however, the speed drops to 0.0 m/s distally towards the tip. This combination (23 Fr. & 1 L/min) has a significantly higher risk for the formation of stasis zones over a wide range of the cannula length.

In the most proximal rows of side holes, symmetrically distributed zones are formed (dashed circle in the right-hand diagram) in which the flow velocity drops to almost 0.0 m/s. This means that even in the immediate inflow area of the side holes there is an area with a potential stasis hazard. This indicates that even in the immediate inflow area of the side holes there is an area with a potential risk of stasis, requiring detailed analysis of which the flow.

A comparison of the LH design variant with the reference design MSC-LH-CI shows that positioning the side holes only near the tip offers no advantage. In the most proximal row, a significantly more pronounced y-profile forms for the flow velocity, which also contains even larger areas of stasis risk. Here too, the velocity decreases distally, although the distribution is more homogeneous in MSC-LH-CI.

**Fig. 6:**
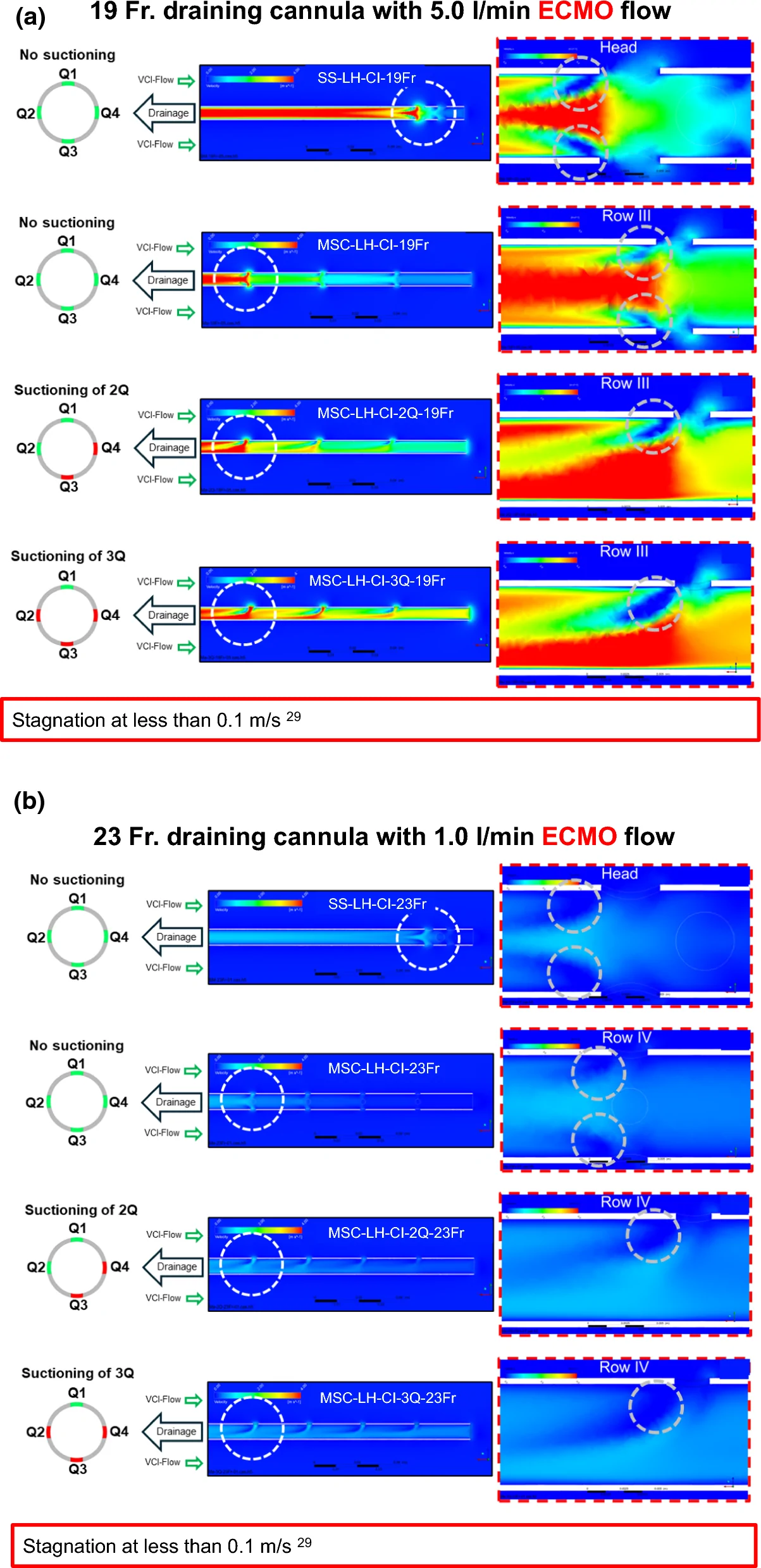
Velocity distribution within cannula for further side holes designs. For the two upper design variants (LH) and (MSC-LH-CI), the side holes are free for all four quadrants. In addition, two quadrants (MSC-LH-CI − 2Q) and three quadrants (MSC-LH-CI − 3Q) are closed for the multi-stage cannula. As before, the highest flow velocity and thus the highest volume flow also occur in the most proximal row (Row III) for these design variants. Distally, the flow velocity decreases and the risk of stasis increases. This is particularly true for the combination of a 23 Fr. cannula and an ECMO flow of only 1.0 L/min. However, it has been shown that closing quadrants has a positive effect on the flow velocity in the distal area of the cannula. The detailed view of Row III or head (dashed circle) shows the formation of symmetrically distributed funnel-shaped zones with velocities of almost 0.0 m/s (dashed circles in the right-hand image). (**A**) Combination of 19 Fr and 5.0 L/min, (**B**) combination of 23 Fr and 1.0 L/min, Velocity range: 0.0 – 4.0 l/min

In reality, a cannula is not centred in the vessel, but may stick to the vessel wall in such a way that the side holes are closed in up to three quadrants in the longitudinal direction (MSC-LH-CI-2Q and MSC-LH-CI-3Q). Remarkably, an improvement in the flow within the cannula can be observed (Fig. 6A, B). This is evident in the fact that the flow velocity within the cannula increases significantly, i.e. the red components increase, and the cannula is better purged due to the increased flow through the cannula tip (Fig. 6A). This significantly reduces the overall area at risk of stasis.

The more quadrants that are no longer available for drainage, the lower the distal reduction in velocity towards the tip. However, this advantage is offset by a more pronounced no-flow zone, which in turn increases the risk of stasis at the proximal side hole row (dashed circle in Fig. 6A, B). The behaviour described above also occurs when combining 23 Fr. and 1.0 L/min, although at a low level. But the proportion is approx. 50% to 60% of the drained volume.

### Flow direction

**Fig. 7:**
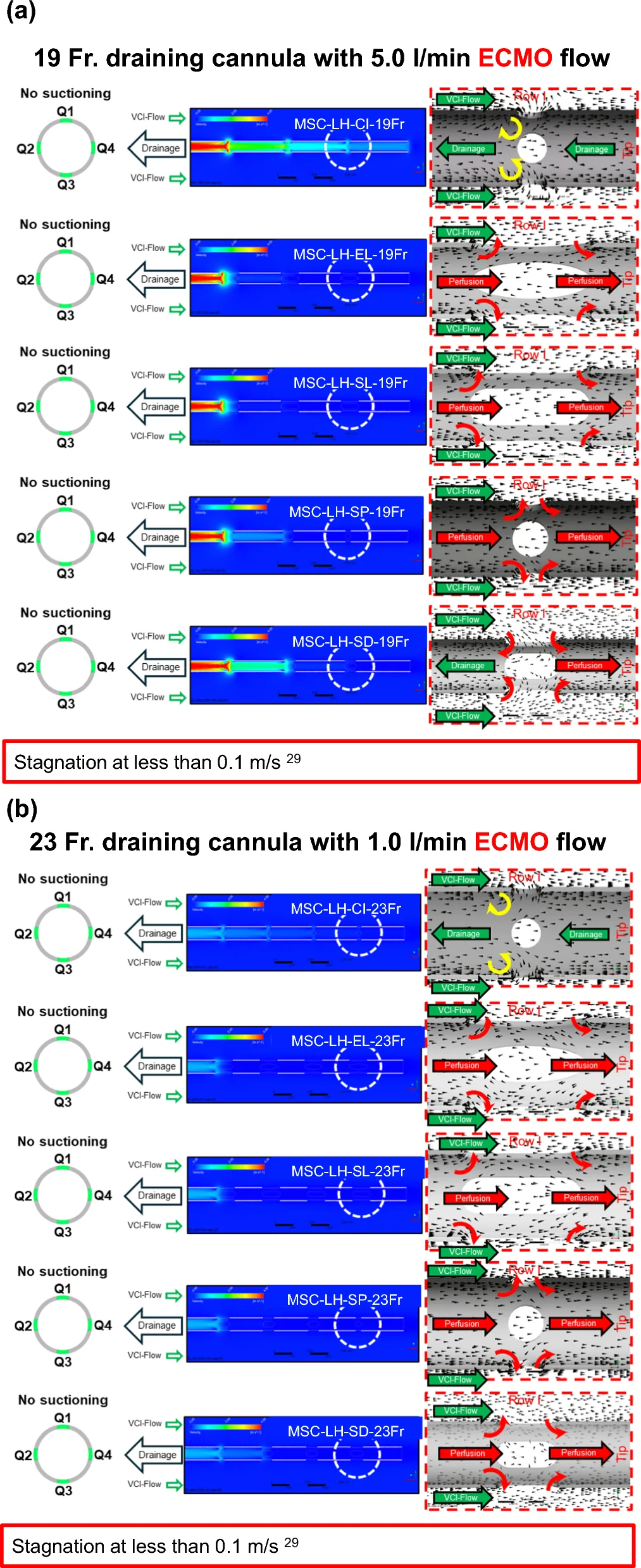
Flow direction for different cannula designs and boundary conditions. For the reference design (multi-staged), there are symmetrically distributed zones with flow velocities of almost 0.0 m/s directly after the side holes. Thrombi can form in these vortices (recirculation areas). In the design variants with a larger surface area of the side holes, blood is perfused from the side holes and the tip into the surrounding area in Row I, although suction is applied. With the MSC-LH-SD variant, on the other hand, only a portion is perfused through the cannula tip. (**A**) Combination of 19 Fr and 5.0 L/min, (**B**) Combination of 23 Fr and 1.0 L/min

In addition to interest in velocity distributions, analysis of the simulations results also focused on flow direction. Row I of side holes was chosen as the location for the analysis, as the lowest velocity level for the side holes can be expected here. The combination of catheter diameter and ECMO that yieded maximum and minimum intra-cannular velocities were selected. Flow was visualised as vectors that only showed the velocity direction (Fig. 7). Arrows indicate the direction of flow. As expected, for the reference design, the flow vectors both before and after the row of side holes Row I point in the proximal direction (green arrows). In contrast, the velocity vectors for the cannulas with enlarged side hole areas point in the distal direction (red arrows). This visualises that no blood is drained through the cannula tip; on the contrary, blood is returned to the vessel. This applied to all diameters and analysed ECMO flows. Only in the MSC-LH-SD design variant did the velocity vectors point in a proximal direction, at least from Row I onwards (Fig. 7A). Below a volume flow of 2.5 L/min or with a cannula diameter greater than 21 Fr., the velocity vectors also point in a distal direction (Fig. 7B).

It should be noted at this point that the volume flow and therefore the flow velocity at which blood exits into the plenum via the tip of the cannula is very low. Furthermore, vector representation of flow velocity in the first row of side holes Row I shows that two counter-rotating vortices (yellow arrows) form in the MSC-LH-CI. Their existence has already been indicated in Fig. 5. In the other design variants MSC-LH-EL, MSC-LH-SL, MSC-LH-SP, however, the lumen is sucked in at the proximal edge and pushed out of the side holes at the distal edge. In the MSC-LH-SD variant, in contrast, lumen is only aspirated at the distal edge of the hole (Fig. 7A).

**Fig. 8:**
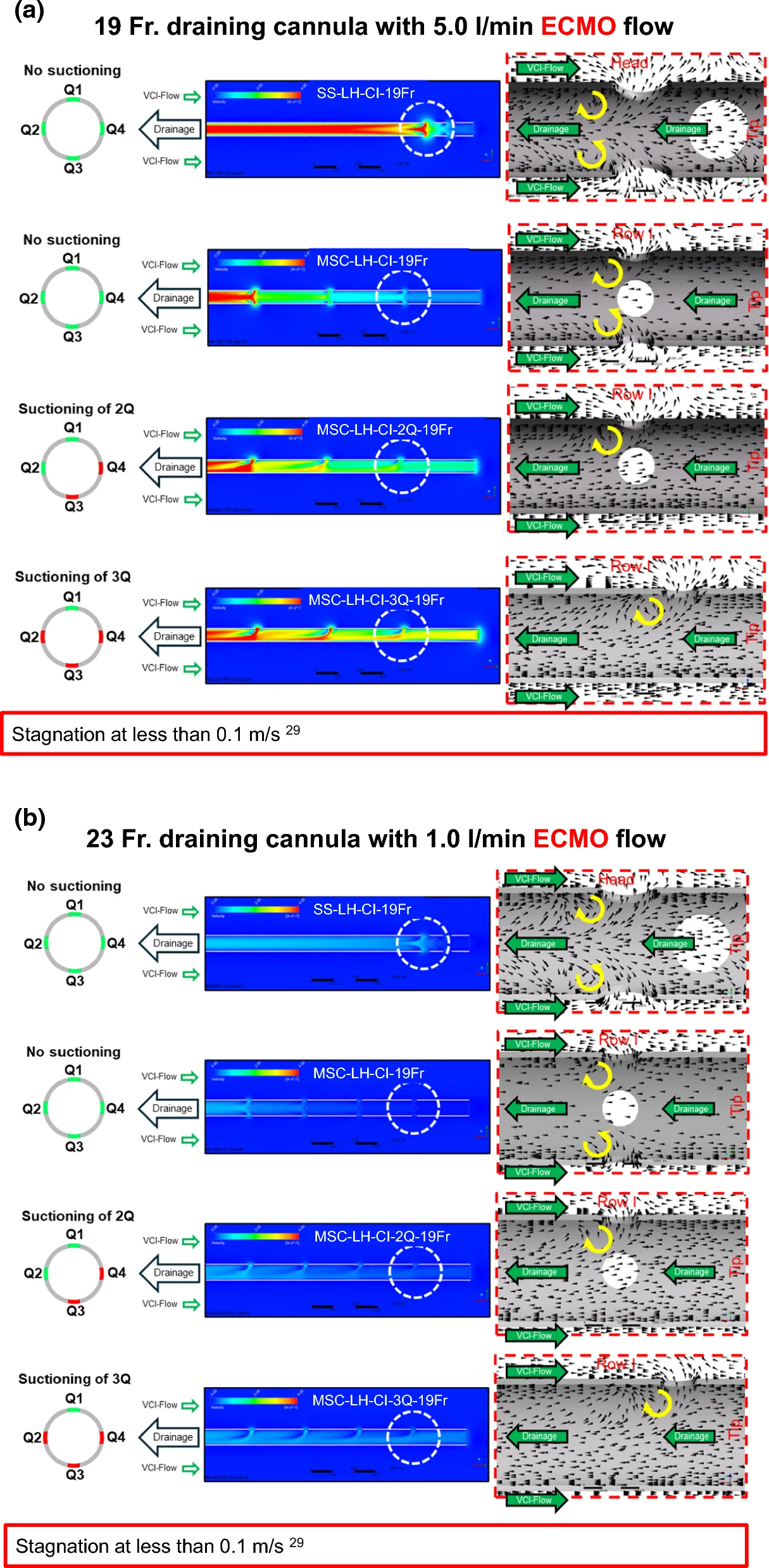
Flow direction for further cannula designs and boundary conditions. In the reference design (multi-staged) of the Lighthouse cannula, there are symmetrically distributed zones directly behind the side holes with flow velocities of almost 0.0 m/s. Their number is directly dependent on the open quadrants. Thrombi can form in these eddies (backflow areas). When the quadrants are closed, recirculation areas also form at the distal side holes (row I), but there is no flow reversal. This means that drainage also occurs through the tip. (**A**) Combination of 19 Fr and 5.0 L/min, (**B**) Combination of 23 Fr and 1.0 L/min

Flow direction during drainage (Fig. 8), in the reference design (MSC-LH-CI) and the Lighthouse design (LH) were in qualitative terms not significantly. Zones with recirculation form both at the concentration of the side holes near the cannula tip (LH) and at the proximal side hole row. Their number and position correspond to the side holes in terms of quadrant and location. Comparing flow direction in closed quadrants (MSC-LH-CI − 2Q and MSC-LH-CI − 3Q) with that in MSC-LH-CI, zones with recirculation also occur, which likewise correspond to the rows of side holes. In contrast to the designs examined previously, for LH, MSC-LH-CI − 2Q and MSC-LH-CI − 3Q, suction is always applied through the tip lumen for both flow diameter combinations. The paradox of perfusion at the tip during drainage was not observed.

Whether degree of recirculation varies for the individual design variants and the residence time of the thrombus-forming components in these areas requires future anaylsis.

### Statistical results

The statistical analyses performed (ANOVA tests) show both significant and insignificant differences when comparing individual side hole rows and the standardised velocity v/v_max_ along the axis of symmetry of the cannulas. Some of these differences are not visible in the figures above. Table 3 below summarises the results of the statistical analysis. The individual rows of side holes are listed in the left-hand column. The comparison partner is shown in the fields themselves. If the fields are highlighted in green, this indicates significance. All other fields are not significant.

**Table 3:** Summary of the results of the statistical analysis using ANOVA tests

Position	MSC-LH-CI-19Fr-05	MSC-LH-EL-19Fr-05	MSC-LH-SL-19Fr-05	MSC-LH-SP-19Fr-05	MSC-LH-SD-19Fr-05
ROW III	Tip	n.s	n.s	n.s	Tip
ROW II	Tip	n.s	ROW I; Tip	n.s	Tip
ROW I	Tip	Tip	Tip	Tip	Tip

Position	MSC-LH-CI-23Fr-01	MSC-LH-EL-23Fr-01	MSC-LH-SL-23Fr-01	MSC-LH-SP-23Fr-01	MSC-LH-SD-23Fr-01
ROW IV	n.s	n.s	n.s	n.s	n.s
ROW III	n.s	ROW II; ROW I; Tip	n.s	n.s	n.s
ROW II	n.s	ROW I; Tip	ROW I; Tip	ROW I; Tip	ROW I; Tip
ROW I	Tip	Tip	Tip	Tip	Tip

Statistical analyses (ANOVA tests) comparing the standardised core speed between different side hole rows and cannulas show some significant but also some insignificant differences, which are not apparent in the figures. All fields without the content ‘n.s.’ indicate significance

## Discussion

The current study reveals flow and stagnation within widely used ECMO cannulas and in those with modified design. The CFD study demonstrates the following:

1. Even with high blood-flow rates up to 5 L/min within 23 Fr. draining cannulas, the most distal part exhibits partial no-flow conditions. This is even more pronounced at lower blood flow rates, often applied in the weaning.
2. Even at the inlet of the side holes, no or very low-flow conditions exist or stagnation and prone to clotting.
3. Configuration and modification of the side holes may improve the observed drawbacks of current cannula design but cannot sufficiently solve them.
4. Reduction of the side holes from 4 quadrants to 2 or 1 in round configurations significantly accelerates the flow at to the tip of the cannula at the expense of increased low flow areas at side hole inlets.
5. Unfavourable combinations of design, flow and diameter can lead to a paradox at the tip of the cannula, i.e. a small section of the lumen is perfused rather than drained.
6. The optimised cannula design improves flow rates in the distal side hole rows and significantly reduces the required suction pressure at high ECMO flow rates and 19 Fr. diameter.

The current data raise some questions about treatment standards and future developments in ECMO therapy. Firstly, there are significant low- and no-flow areas within an ECMO cannula, generally located at the tip of the cannula and slightly below the entry point of the blood at the side holes. Both points are design sites likely to result in clotting, which may lead to further clotting complications within the blood pump, haemolysis and bleeding, all likely to have a serious impact on patients’ outcomes. Anticoagulation and high blood flow rates may counteract this feature, but in daily clinical practice fluctuations occur in both dimensions. Furthermore, during weaning off ECMO, many medical centres reduce the blood flow rate and therefore may exacerbate clotting at these sites. Our data concur with other reported simluations [14, 16–18] pointing out the importance of new cannula [30] designs that mitigate against the occurrence of low-flow areas.

Our simulation of various design options, with variations in the cross-sectional area of the side holes showed an overall improvement. However, velocity at the cannula tip was still low. Examining the suction of the cannula on the vessel wall, which can lead to complete occlusion of the entire lateral perfusion of one or more quadrants, surprisingly showed a significant improvement in velocity and flow distribution on both cases. However, improving the flow velocity at the lateral openings leads to a slightly larger area with low flow below the blood entry point, and problems at the cannula tip may be overcome with this configuration. Of note, anticoagulation cannot fully reverse the effects of the artificial surface and the major drawbacks of current cannulas.

### Suction flow partition

The ECMO blood pump generates a pressure difference to drain a defined volume flow *Q* through the cannula. The side holes in such a cannula are intended to ensure continued drainage as a safety reserve in the event of the cannula tip becoming blocked. However, any additional opening (side holes) to the cannula tip means a pressure drop at these openings.

Similar to findings reported by Rauh et al. [17], Broman [16], Vatani et al. [14] and Fiusco et al. [18] this study shows that for cannula designs with circular side holes, the greatest pressure reduction occurs at the proximally located side holes. With each additional distal row of side holes, the pressure difference as the driving force for flow decreases. The flow data in Table 2 for the three sizes of the reference cannula impressively demonstrate the reduction in percentage flow for Row III/IV, which decreases from 56.7% to 42.5% as the diameter increases. Pressure reduction phenomenon occurs without exception for all cannula diameters and volume flows (Supplement C).

### Flow pattern

The larger surface areas of the side holes reduce the overall pressure loss, but also decrease the flow velocity distal to the side holes within the cannula. The contrasting effects of the side holes themselves and their surface areas are reflected not only in the tables and diagrams, but also very clearly in the representation of the velocity distribution and velocity vectors within the cannula.

Analysis of the flow behaviour using velocity vectors reveals even paradoxical behaviour between the proximal first row of side holes (Row III and Row IV) and Row I and the cannula tip (Fig. 7A, B). Although this entire constellation involves drainage, a zone of perfusion occurs, i.e. some of the lumen contents is returned to the blood vessel through the side holes and the tip, a feature critical for possible side effects such as clotting. The pressure difference between the vessel and the cannula in this area is in the single-digit Pascal range, so that part of the fluid volume sucked in proximally can move distally. Furthermore, the flow surrounding the cannula is sucked back into the blood vessel from the VCI lumen, as in a Venturi nozzle. However, the velocities are in the range of approximately 1.0 to 7.0 mm/s, nearby stagnation in these areas.

As shown by Vatani et al. [14], reducing the area of the side holes proximally maybe an approach to overcome this problem. However, it should be noted that several parameters were changed in combination and that a greatly simplified geometry of the right atrium was used, so that the observed effects cannot be clearly attributed to the parameters.

### Limitations

**Fig. 9:**
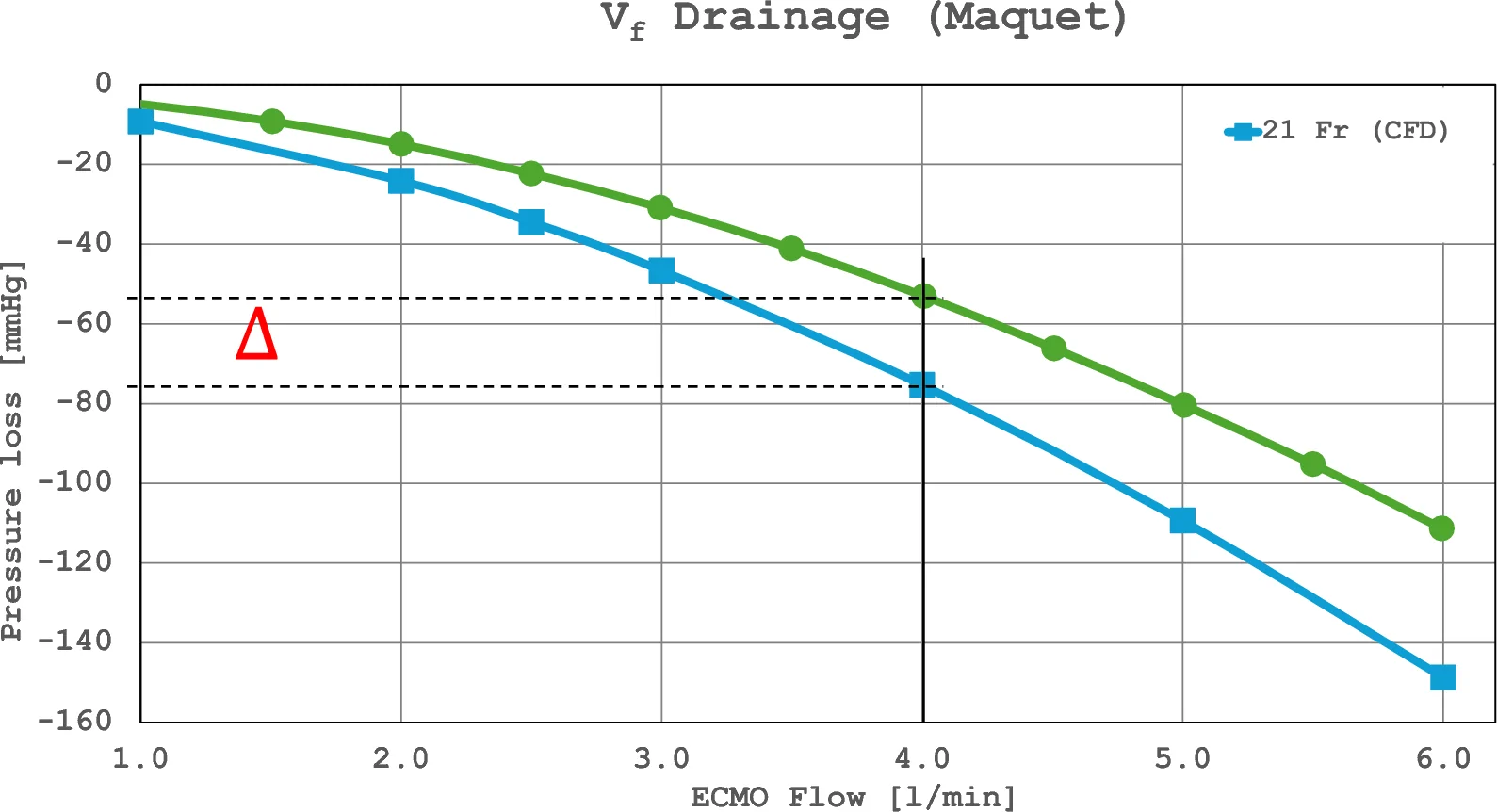
Limited comparability of the pressure loss curves. The measured and the simulated pressure loss curve are only qualitatively comparable for all operating points of the cannula design. The comparison shows the absolute difference. This is due to the unknown boundary conditions of the measurements

An elementary basis for comparison is that the boundary conditions for measurement and simulation are identical. The manufacturer's specifications for the Maquet^©^ reference cannula were used to compare the calculated pressure loss curves [30]. A comparison of the two curves reveals significant deviation for a flow rate of 4.0 L/min, for example Fig. 9. Due to the large deviation, it can be assumed that there are differences not only in the plenum used but also in the fluid. For the simulation, the dynamic viscosity of blood as a function of the shear rate was used (Eq. 2). With blood, pressure losses can be between 10 and 350% higher than with water [31]. This leaves only the tendency, i.e., the qualitative comparison of the pressure loss curves, which is consistent for both cannulas.

Various research groups have described the phenomenon of widely varying drainage performance. In the work by Rauh et al. [17], the simulation results for a 25 Fr. cannula from Getinge© were validated with measurements in 4D MRI. However, these confirmed values can only be used for *qualitative* validation of the simulation results of this study, as direct comparison is not possible despite the same cannula due to different boundary conditions. The main differences are:

- Taking into account the flow from the inferior vena cava (VCI) (in this study) or superior vena cava (VCS) in ^17^. However, whether neglecting the volume flow from the VCS is permissible requires confirmation in further studies.
- The volume of fluid used (VC vs. plenum). When using a solid plastic tube as a vein model, interactions occur between proximately opposed walls, which influence the overall velocity and pressure distribution.
- The use of Newtonian fluid water in [17] $\left(\mu =const.\right)$ vs. the consideration of dynamic viscosity $\mu =f\left(\dot{\gamma }\right)$ due to the non-Newtonian properties of blood in this study.

Nevertheless, it can be seen that the difference between the numerical values is relatively small in some cases, such as in row IV and at the tip of the cannula (Supplementary Table 11).

## Conclusions

The role of the draining cannula design on clotting seems to be largely underestimated in current ECMO technology. Low-flow and no-flow areas are prone for clotting complications and should be a future target for new cannula designs. Furthermore, clinicians should be aware that low blood flow rates even pronounce this problem and should carefully monitor coagulation parameter and complications during the weaning process. Modified designs decrease the suction pressure to reduce the risk of haemolysis while at the same time moderately improving the overall flow pattern within the cannula with terms of the inclination to clotting.

## Take-home-message

Cannulas are the direct and indispensible interface between patients and extracorporeal membrane oxygenation but may also cause complications such as clotting. The current study shows relevant low-flow areas within typical draining cannulas for VV-ECMO and VA-ECMO treatment and evaluates new beneficial design options. In general much more attention should be paid to the design aiming on reducing stagnation areas within cannulas.

## Supplementary Information

Additional file 1

## Data Availability

Not applicable.

## Abbreviations

AvWS: Acquired van-willebrand syndrom
BC: Boundary condition
CAD: Computer-aided design
CFD: Computational fluid dynamics
CO: Cardiac output
CVP: Central venous pressure
Fr: French
HKT: Haematocrit
MSC LH-CI: Lighthouse multi-staged continuous circle
MSC LH-CI-2Q: Lighthouse multi-stagedcontinuous circle (2 lines closed)
MSC LH-CI-3Q: Lighthouse multi-staged continuous circle (3 lines closed)
MSC LH-EL: Lighthouse multi-staged continuous elliptical
MSC LH-SD: Lighthouse multi-staged continuous slothole distal
MSC LH-SL: Lighthouse multi-staged continuous slothole
MSC LH-SP: Lighthouse multi-staged continuous slothole proximal
SS-LH-CI: Lighthouse single-staged, circle
SST: Shear stress transport
VA-ECMO: Veno-arterial extra corporeal membrane oxygenation
VCI: Vena cava inferior
VCS: Vena cava superior
VV-ECMO: Veno-venous extra corporeal membrane oxygenation
WSS: Wall shear stress
