# Supplementary material for: Impact of side hole design of drainage cannulas on flow velocity and predilection sites for low-flow areas in veno-venous ECMO circuits
Source: Intensive Care Med Exp. 2026 Oct 9;14:130. doi: 10.1186/s40635-026-00980-8 (PMC13653020; doi:10.1186/s40635-026-00980-8)
Supplement: Supplementary file 1 [file 40635_2026_980_MOESM1_ESM.docx]

# Supplement A

## Pressure loss

In order to drain blood from the body at a rate of, for example, 5 l/min, the blood pump must generate a correspondingly high difference in pressure between the ambient pressure and the CVP (see tables in Supplement A). This pressure causes mechanical overstressing of the cell membranes of the erythrocytes due to the high shear forces that occur, leading to their dissolution and thus to haemolysis.

The standard Maquet® cannula with the three most frequently used diameters 19 Fr, 21 Fr and 23 Fr was used as a reference for the quantitative assessment of the influence of the different design variants of the side holes. Figure 10A shows exemplarily the pressure loss curves over the ECMO flow for the 23 Fr. variants. All four side hole variants show significantly better behaviour in terms of pressure loss. They all lie above the cannula with the circular side holes. The slot hole variant (MSC-LH-SL) has the lowest pressure loss over the entire range. The design of the Slothole proximal cannula (MSC-LH-SP) is almost identical to this. The cannula with the elliptical side holes (MSC-LH-EL) has a slightly lower pressure loss. Although the distal slot hole design (MSC-LH-SD) is closest to the MSC-LH-CI cannula, it still results in a lower pressure loss curve. The pressure loss curves apply to all diameters analysed. A multi-staged cannula that is sucked against the vessel wall, i.e. the side holes of two or three quadrants are closed, has the same pressure loss behaviour as a free-aspirating lighthouse cannula. However, in this situation it has a worse pressure loss curve than the multi-staged cannula that drains freely for all four quadrants (Figure 10B).

## Percentage drainage flow

The names of the various cannulas are briefly explained in Table 1. The two digits that follow indicate the cannula diameter.

In the MSC-LH-SL design variant, the surface area of the side holes has been increased by 471.85% compared to the reference design. If we now look at the percentage of side holes in the total aspirated lumen for this design (Supplementary Table 4), the drainage behaviour has changed drastically. On average, 99% to 95% are aspirated from the proximal row of side holes. The next distal row of side holes contributes only a marginal 0.8% to 3.9% of the suction. All other rows of side holes and the tip of the cannula drain a maximum of 0.4% of the plenum. The cannula with the elliptical side holes exhibits identical suction behaviour.

Based on the above result, the area of the side holes has been reduced exponentially with each side hole row distal from slot hole to round (variant MSC-LH-SP). As a result, the total area of the side holes has increased by 170.75% (19 Fr., 21 Fr.) and 236.6% (23 Fr.) compared to the reference design. However, it was also shown here that drainage occurs almost exclusively in the proximal row of side holes (Supplementary Table 5).

The percentage flow share of the proximal rows is only marginally better than for the other design variants MSC-LH-EL (Supplementary Table 6) and MSC-LH-SL (Supplementary Table 4).

As a further variant, the area of the side holes has been reduced exponentially with each row proximally from slot hole to round. With regard to the total area, there is no difference to the MSC-LH-SP variant. However, when looking at the percentage of the total drainage volume (Supplementary Table 7), the difference between this MSC-LH-SD variant and the previous design variants becomes clear. Although the largest proportion of flow (on average 66.9% to 53.2%) is aspirated through the proximal side holes, as with all variants, it does not drop rapidly in the next distal row of side holes. The percentage flow share is almost halved as in the reference design. Only then does it drop sharply to between 0.1% and 0.3%. A comparison of the two cannula diameters 19 Fr. and 21 Fr. with their three rows of side holes shows that the mean values of their percentage shares only differ between 0.1% and 6.6% for the respective rows of side holes.

Supplementary Table 4: Slot hole design (MSC-LH-SL): Operating data and drainage performance

|  |  |  |  | Flow | | | | |
| --- | --- | --- | --- | --- | --- | --- | --- | --- |
| Cannula | Operating flow [L/min] | Pressure loss [mmHg] | max. Velocity [m/s] | (Row IV) [%] | (Row III) [%] | (Row II) [%] | (Row I) [%] | (Tip) [%] |
| MSC-LH-SL-19 | 1,0 | -13,0 | 1,1 |  | 98,9 | 1,2 | 0,1 | 0,2 |
|  | 2,0 | -35,1 | 2,1 |  | 99,7 | 0,4 | 0,0 | 0,1 |
|  | 2,5 | -50,5 | 2,6 |  | 100,1 | 0,0 | 0,0 | 0,1 |
|  | 3,0 | -68,2 | 3,1 |  | 99,9 | 0,2 | 0,0 | 0,1 |
|  | 4,0 | -109,8 | 4,1 |  | 99,2 | 0,8 | 0,0 | 0,1 |
|  | 5,0 | -159,5 | 5,0 |  | 98,6 | 1,4 | 0,1 | 0,1 |
|  | 6,0 | -217,4 | 6,0 |  | 98,2 | 1,7 | 0,1 | 0,0 |
| MSC-LH-SL-21 | 1,0 | -8,4 | 0,9 |  | 98,9 | 1,2 | 0,1 | 0,3 |
|  | 2,0 | -21,8 | 1,7 |  | 100,0 | 0,1 | 0,0 | 0,1 |
|  | 2,5 | -31,2 | 2,1 |  | 99,4 | 0,7 | 0,00 | 0,1 |
|  | 3,0 | -42,3 | 2,5 |  | 98,7 | 1,4 | 0,1 | 0,1 |
|  | 4,0 | -68,5 | 3,2 |  | 97,5 | 2,4 | 0,2 | 0,1 |
|  | 5,0 | -99,6 | 4,1 |  | 96,9 | 3,0 | 0,2 | 0,1 |
|  | 6,0 | -135,7 | 5,0 |  | 96,4 | 3,4 | 0,2 | 0,1 |
| MSC-LH-SL-23 | 1,0 | -5,8 | 0,7 | 99,2 | 0,9 | 0,2 | 0,3 | 0,5 |
|  | 2,0 | -14,9 | 1,4 | 97,9 | 2,1 | 0,3 | 0,1 | 0,3 |
|  | 2,5 | -20,8 | 1,7 | 96,4 | 3,3 | 0,5 | 0,1 | 0,2 |
|  | 3,0 | -28,1 | 2,0 | 95,4 | 4,2 | 0,6 | 0,0 | 0,2 |
|  | 4,0 | -45,6 | 2,8 | 94,5 | 5,1 | 0,6 | 0,0 | 0,2 |
|  | 5,0 | -66,6 | 3,5 | 93,9 | 5,7 | 0,5 | 0,0 | 0,1 |
|  | 6,0 | -90,9 | 4,2 | 93,6 | 6,0 | 0,5 | 0,0 | 0,1 |

The slot hole (MSC-LH-SL) design variant shows a completely different behaviour compared to the reference design. Between 95% and 99% of the total drainage volume is extracted through the proximal row of side holes (Row III or Row IV). Accordingly, all other side holes contribute very little.

Supplementary Table 5: Slot hole proximal (MSC-LH-SP): Operating Data and drainage performance

|  |  |  |  | Flow | | | | |
| --- | --- | --- | --- | --- | --- | --- | --- | --- |
| Cannula | Operating flow [L/min] | Pressure loss [mmHg] | max. Velocity [m/s] | (Row IV) [%] | (Row III) [%] | (Row II) [%] | (Row I) [%] | (Tip) [%] |
| MSC-LH-SP-19 | 1,0 | -13,0 | 1,1 |  | 98,0 | 2,0 | 0,1 | 0,1 |
|  | 2,0 | -35,3 | 2,1 |  | 94,5 | 5,5 | 0,1 | 0,1 |
|  | 2,5 | -50,9 | 2,6 |  | 93,5 | 6,4 | 0,1 | 0,1 |
|  | 3,0 | -68,7 | 3,1 |  | 92,9 | 7,0 | 0,1 | 0,1 |
|  | 4,0 | -110,7 | 4,1 |  | 92,1 | 7,8 | 0,1 | 0,1 |
|  | 5,0 | -160,8 | 5,0 |  | 91,7 | 8,3 | 0,1 | 0,1 |
|  | 6,0 | -219,2 | 6,0 |  | 91,4 | 8,6 | 0,0 | 0,0 |
| MSC-LH-SP-21 | 1,0 | -8,5 | 0,9 |  | 94,9 | 5,2 | 0,1 | 0,1 |
|  | 2,0 | -22,1 | 1,7 |  | 90,9 | 9,1 | 0,1 | 0,1 |
|  | 2,5 | -31,6 | 2,1 |  | 90,0 | 10,0 | 0,1 | 0,1 |
|  | 3,0 | -42,7 | 2,5 |  | 89,4 | 10,6 | 0,1 | 0,1 |
|  | 4,0 | -69,2 | 3,3 |  | 88,7 | 11,3 | 0,0 | 0,0 |
|  | 5,0 | -100,6 | 4,1 |  | 88,1 | 11,7 | 0,2 | 0,0 |
|  | 6,0 | -137,1 | 5,0 |  | 87,7 | 11,9 | 0,4 | 0,0 |
| MSC-LH-SP-23 | 1,0 | -5,9 | 0,7 | 93,4 | 6,6 | 0,1 | 0,1 | 0,2 |
|  | 2,0 | -14,9 | 1,4 | 89,0 | 10,7 | 0,4 | 0,0 | 0,2 |
|  | 2,5 | -20,9 | 1,7 | 88,3 | 11,5 | 0,3 | 0,0 | 0,1 |
|  | 3,0 | -28,2 | 2,0 | 87,9 | 12,0 | 0,2 | 0,0 | 0,1 |
|  | 4,0 | -45,9 | 2,8 | 87,3 | 12,6 | 0,1 | 0,0 | 0,1 |
|  | 5,0 | -67,1 | 3,5 | 86,7 | 12,8 | 0,5 | 0,0 | 0,1 |
|  | 6,0 | -91,5 | 4,2 | 86,2 | 12,9 | 0,9 | 0,0 | 0,1 |

Summary of the percentage shares of the respective side hole rows in the extracted volume flow for the cannula with the exponentially reduced side holes (variant MSC-LH-SP). This confirms the phenomenon that drainage occurs almost exclusively in the proximal row of side holes.

Supplementary Table 6: Slot hole elliptical (MSC-LH-EL): Operating Data and drainage performance

|  |  |  |  | Flow | | | | |
| --- | --- | --- | --- | --- | --- | --- | --- | --- |
| Cannula | Operating flow [L/min] | Pressure loss [mmHg] | max. Velocity [m/s] | (Row IV) [%] | (Row III) [%] | (Row II) [%] | (Row I) [%] | (Tip) [%] |
| MSC-LH-EL-19 | 1,0 | -13,2 | 1,1 |  | 99,4 | 0,7 | 0,1 | 0,2 |
|  | 2,0 | -35,9 | 2,1 |  | 99,7 | 0,4 | 0,0 | 0,1 |
|  | 2,5 | -51,7 | 2,6 |  | 99,0 | 1,0 | 0,0 | 0,1 |
|  | 3,0 | -69,7 | 3,1 |  | 98,4 | 1,6 | 0,1 | 0,1 |
|  | 4,0 | -112,0 | 4,0 |  | 97,6 | 2,4 | 0,1 | 0,1 |
|  | 5,0 | -162,8 | 5,0 |  | 97,1 | 2,9 | 0,1 | 0,1 |
|  | 6,0 | -222,0 | 6,0 |  | 96,7 | 3,2 | 0,1 | 0,0 |
| MSC-LH-EL-21 | 1,0 | -8,6 | 0,9 |  | 99,9 | 0,2 | 0,1 | 0,2 |
|  | 2,0 | -22,3 | 1,7 |  | 98,1 | 2,0 | 0,0 | 0,1 |
|  | 2,5 | -31,9 | 2,1 |  | 97,0 | 3,0 | 0,1 | 0,1 |
|  | 3,0 | -43,2 | 2,5 |  | 96,2 | 3,7 | 0,2 | 0,1 |
|  | 4,0 | -69,9 | 3,3 |  | 95,3 | 4,6 | 0,2 | 0,1 |
|  | 5,0 | -101,8 | 4,1 |  | 94,7 | 5,1 | 0,3 | 0,1 |
|  | 6,0 | -138,8 | 5,0 |  | 94,4 | 5,5 | 0,2 | 0,1 |
| MSC-LH-EL-23 | 1,0 | -5,9 | 0,7 | 99,0 | 1,1 | 0,1 | 0,2 | 0,5 |
|  | 2,0 | -15,2 | 1,4 | 94,1 | 5,5 | 0,6 | 0,1 | 0,3 |
|  | 2,5 | -21,2 | 1,7 | 93,1 | 6,5 | 0,6 | 0,1 | 0,2 |
|  | 3,0 | -28,6 | 2,1 | 92,4 | 7,1 | 0,6 | 0,0 | 0,2 |
|  | 4,0 | -46,6 | 2,8 | 91,7 | 7,9 | 0,6 | 0,0 | 0,1 |
|  | 5,0 | -68,1 | 3,5 | 91,3 | 8,3 | 0,5 | 0,0 | 0,1 |
|  | 6,0 | -93,0 | 4,2 | 91,1 | 8,7 | 0,4 | 0,0 | 0,1 |

Summary of the percentage shares of the respective side hole rows in the aspirated volume flow for the cannula with the elliptical side holes (variant MSC-LH-EL). As with the MSC-LH-SL and MSC-LH-SP variants, hardly any blood is aspirated from the plenum in the distal rows of side holes.

Supplementary Table 7: Slot hole distal (MSC-LH-SD): Operating Data and drainage performance

|  |  |  |  | Flow | | | | |
| --- | --- | --- | --- | --- | --- | --- | --- | --- |
| Cannula | Operating flow [L/min] | Pressure loss [mmHg] | max. Velocity [m/s] | (Row IV) [%] | (Row III) [%] | (Row II) [%] | (Row I) [%] | (Tip) [%] |
| MSC-LH-SD-19 | 1,0 | -13,5 | 1,1 |  | 70,2 | 29,7 | 0,3 | 0,2 |
|  | 2,0 | -36,6 | 2,1 |  | 68,0 | 31,9 | 0,2 | 0,1 |
|  | 2,5 | -52,8 | 2,6 |  | 67,3 | 32,1 | 0,7 | 0,1 |
|  | 3,0 | -71,3 | 3,1 |  | 66,8 | 32,2 | 1,27 | 0,1 |
|  | 4,0 | -114,7 | 4,1 |  | 65,9 | 32,3 | 2,0 | 0,1 |
|  | 5,0 | -166,9 | 5,1 |  | 65,3 | 32,4 | 2,5 | 0,2 |
|  | 6,0 | -227,7 | 6,0 |  | 64,9 | 32,5 | 2,8 | 0,2 |
| MSC-LH-SD-21 | 1,0 | -8,8 | 0,9 |  | 63,9 | 36,2 | 0,2 | 0,2 |
|  | 2,0 | -22,9 | 1,7 |  | 61,4 | 36,8 | 2,0 | 0,2 |
|  | 2,5 | -32,7 | 2,1 |  | 60,7 | 36,7 | 2,9 | 0,2 |
|  | 3,0 | -44,3 | 2,5 |  | 60,1 | 36,6 | 3,6 | 0,3 |
|  | 4,0 | -71,6 | 3,2 |  | 59,3 | 36,5 | 4,5 | 0,3 |
|  | 5,0 | -104,0 | 4,0 |  | 58,7 | 36,6 | 5,0 | 0,3 |
|  | 6,0 | -141,6 | 4,8 |  | 58,2 | 36,6 | 5,4 | 0,2 |
| MSC-LH-SD-23 | 1,0 | -6,0 | 0,7 | 56,5 | 41,7 | 2,0 | 0,3 | 0,5 |
|  | 2,0 | -15,5 | 1,4 | 54,2 | 40,3 | 5,7 | 0,1 | 0,3 |
|  | 2,5 | -21,7 | 1,7 | 53, | 40,0 | 6,6 | 0,2 | 0,3 |
|  | 3,0 | -29,2 | 2,0 | 53,0 | 39,9 | 7,2 | 0,2 | 0,3 |
|  | 4,0 | -47,5 | 2,6 | 52,3 | 39,8 | 8,0 | 0,1 | 0,2 |
|  | 5,0 | -69,3 | 3,3 | 51,9 | 39,9 | 8,4 | 0,0 | 0,2 |
|  | 6,0 | -94,4 | 3,9 | 51,4 | 39,9 | 8,7 | 0,2 | 0,1 |

The design (MSC-LH-SD) with the proximally reduced surfaces of the side holes causes the proximally located row of side holes (Row III or Row IV) to aspirate between 53% and 67% of the total volume. This proportion is almost reduced by half in the next distal row of side holes (Row II or Row III). Only then does it drop rapidly towards zero.

Due to the fact that in this study the analysis of the previous design variants was only carried out for extreme operating conditions, this was done for both the Lighthouse single-staged cannula and the situation of a suctioned multi-staged cannula.

As with all other variants, the single-stage arrangement of the side holes shows that the proximal row of side holes contributes the largest share to drainage, decreasing distally. The six side holes are summarised in Supplementary Table 8 in Row I (93.51%). A proportion of 6.28% is aspirated through the tip. The numerical values for rows II to IV are based on mathematical inaccuracies and are negligible. Compared to the combination of 23 Fr. with 1.0 L/min, drainage is improved by the tip

Supplementary Table 8: Lighthouse single-staged (LH): Operating Data and drainage performance

|  |  |  |  | Flow | | | | |
| --- | --- | --- | --- | --- | --- | --- | --- | --- |
| Cannula | Operating flow [L/min] | Pressure loss [mmHg] | max. Velocity [m/s] | (Row IV) [%] | (Row III) [%] | (Row II) [%] | (Row I) [%] | (Tip) [%] |
| SS-LH-CI-19 | 1,0 | -16,2 | 1,2 |  | 0,2 | 0,2 | 105,1 | 5,4 |
|  | 2,0 | -44,6 | 2,2 |  | 0,2 | 0,1 | 98,7 | 1,0 |
|  | 2,5 | -64,0 | 2,7 |  | 0,1 | 0,1 | 97,2 | 2,6 |
|  | 3,0 | -86,4 | 3,2 |  | 0,1 | 0,1 | 96,0 | 3,7 |
|  | 4,0 | -138,5 | 4,3 |  | 0,1 | 0,1 | 94,5 | 5,3 |
|  | 5,0 | -200,5 | 5,3 |  | 0,1 | 0,1 | 93,5 | 6,3 |
|  | 6,0 | -272,8 | 6,3 |  | 0,1 | 0,1 | 92,8 | 7,0 |
| SS-LH-CI-23 | 1,0 | -7,1 | 0,8 | 0,0 | 0,1 | 0,0 | 89,8 | 10,1 |
|  | 2,0 | -18,2 | 1,5 | 0,0 | 0,1 | 0,0 | 86,0 | 13,9 |
|  | 2,5 | -25,9 | 1,8 | 0,0 | 0,1 | 0,0 | 85,0 | 15,0 |
|  | 3,0 | -35,1 | 2,1 | 0,0 | 0,1 | 0,0 | 84,2 | 15,8 |
|  | 4,0 | -56,4 | 2,8 | 0,0 | 0,1 | 0,0 | 83,1 | 16,8 |
|  | 5,0 | -81,7 | 3,5 | 0,0 | 0,1 | 0,0 | 82,4 | 17,5 |
|  | 6,0 | -110,9 | 4,3 | 0,0 | 0,1 | 0,0 | 81,9 | 18,0 |

The single-stage arrangement of the side holes (LH), i.e. their concentrated positioning just behind the tip, ensures that the greatest drainage performance occurs precisely there. Nevertheless, up to 10% of the lumen is extracted through the tip.

Contrary to expectations that complete closure of two (Supplementary Table 9) or more quadrants would impair the drainage performance of the cannula, it improved. Although the percentage of drained lumen also decreased distally, this was only by approximately 10% in each row. It is noteworthy that the percentage through the tip rises again to almost the value of the most proximal side hole row. In the case examined, where suction is only possible through one quadrant (Supplementary Table 10), the percentages change such that approximately 50% to 60% is suctioned through the tip of the cannula. The remaining percentages decrease again distally.

Supplementary Table 9: Reference design with 2 quadrants closed (MSC-LH-CI-2Q): Operating Data and drainage performance

|  |  |  |  | Flow | | | | |
| --- | --- | --- | --- | --- | --- | --- | --- | --- |
| Cannula | Operating flow [L/min] | Pressure loss [mmHg] | max. Velocity [m/s] | (Row IV) [%] | (Row III) [%] | (Row II) [%] | (Row I) [%] | (Tip) [%] |
| MSC-LH-CI-2Q-19 | 1,0 | -15,6 | 1,2 |  | 41,1 | 25,6 | 14,7 | 18,5 |
|  | 2,0 | -42,8 | 2,3 |  | 37,8 | 24,2 | 14,6 | 23,4 |
|  | 2,5 | -61,2 | 2,8 |  | 37,0 | 23,8 | 14,6 | 24,6 |
|  | 3,0 | -82,4 | 3,3 |  | 36,3 | 23,6 | 14,6 | 25,6 |
|  | 4,0 | -132,3 | 4,3 |  | 35,4 | 23,1 | 14,6 | 27,0 |
|  | 5,0 | -192,3 | 5,3 |  | 34,7 | 22,9 | 14,6 | 27,9 |
|  | 6,0 | -262,5 | 6,3 |  | 34,1 | 22,7 | 14,6 | 28,6 |
| MSC-LH-CI-2Q-23 | 1,0 | -6,9 | 0,8 | 28,0 | 20,4 | 14,4 | 9,5 | 27,8 |
|  | 2,0 | -18,1 | 1,5 | 25,9 | 19,2 | 14,0 | 9,7 | 31,2 |
|  | 2,5 | -25,5 | 1,9 | 25,3 | 18,9 | 13,9 | 9,7 | 32,2 |
|  | 3,0 | -34,2 | 2,2 | 24,9 | 18,7 | 13,8 | 9,8 | 32,9 |
|  | 4,0 | -55,1 | 2,9 | 24,3 | 18,3 | 13,6 | 9,8 | 34,0 |
|  | 5,0 | -80,2 | 3,6 | 23,9 | 18,0 | 13,5 | 9,8 | 34,8 |
|  | 6,0 | -109,1 | 4,3 | 23,6 | 17,9 | 13,4 | 9,9 | 35,3 |

The closure of two quadrants at side hole rows (MSC-LH-CI-2Q) improves drainage performance in that the percentage decreases distally but rises again to almost the value of the most proximal row at the cannula tip.

Supplementary Table 10: Reference design with 3 quadrants closed (MSC-LH-C -2Q): Operating Data and drainage performance

|  |  |  |  | Flow | | | | |
| --- | --- | --- | --- | --- | --- | --- | --- | --- |
| Cannula | Operating flow [L/min] | Pressure loss [mmHg] | max. Velocity [m/s] | (Row IV) [%] | (Row III) [%] | (Row II) [%] | (Row I) [%] | (Tip) [%] |
| MSC-LH-CI-3Q-19 | 1,0 | -16,1 | 1,4 |  | 24,4 | 18,3 | 12,9 | 44,5 |
|  | 2,0 | -44,3 | 2,5 |  | 21,8 | 16,8 | 12,4 | 49,0 |
|  | 2,5 | -63,2 | 3,0 |  | 21,1 | 16,4 | 12,3 | 50,2 |
|  | 3,0 | -84,8 | 3,6 |  | 20,7 | 16,1 | 12,2 | 51,0 |
|  | 4,0 | -136,4 | 4,6 |  | 20,1 | 15,7 | 12,0 | 52,1 |
|  | 5,0 | -199,3 | 5,7 |  | 19,9 | 15,6 | 11,9 | 52,7 |
|  | 6,0 | -272,1 | 6,8 |  | 19,6 | 15,4 | 11,9 | 53,1 |
| MSC-LH-CI-3Q-23 | 1,0 | -7,1 | 0,9 | 15,5 | 12,7 | 10,2 | 7,9 | 53,8 |
|  | 2,0 | -18,3 | 1,6 | 14,0 | 11,6 | 9,6 | 7,8 | 57,0 |
|  | 2,5 | -25,9 | 2,0 | 13,6 | 11,4 | 9,5 | 7,8 | 57,9 |
|  | 3,0 | -34,6 | 2,3 | 13,3 | 11,1 | 9,4 | 7,8 | 58,5 |
|  | 4,0 | -55,3 | 3,0 | 12,9 | 10,9 | 9,2 | 7,8 | 59,3 |
|  | 5,0 | -80,7 | 3,7 | 12,8 | 10,8 | 9,1 | 7,7 | 59,7 |
|  | 6,0 | -110,2 | 4,4 | 12,6 | 10,7 | 9,1 | 7,7 | 59,9 |

The closure of three quadrants at rows of side holes (MSC-LH-CI-3Q) improves drainage performance in that the percentage drops distally but rises again to 50% to 60% of the aspirated volume flow at the cannula tip.

Supplementary Table 11: Drainage performance across all relevant volume flows of a 25 Fr. cannula from Maquet©

|  | ECMO flow [L/min] | Flow fraction (Row V) [%] | | Flow fraction (Row IV) [%] | | Flow fraction (Row III) [%] | | Flow fraction (Row II) [%] | | Flow fraction (Row I) [%] | | Flow fraction (Tip) [%] | |
| --- | --- | --- | --- | --- | --- | --- | --- | --- | --- | --- | --- | --- | --- |
| MSC-LH-CI-25 |  | Rauh et al | Bongert et al | Rauh et al | Bongert et al | Rauh et al | Bongert et al | Rauh et al | Bongert et al | Rauh et al | Bongert et al | Rauh et al | Bongert et al |
|  | 1.0 | 49.7 | 39.3 | 24.4 | 24.8 | 11.8 | 15.3 | 5.0 | 9.0 | 1.0 | 4.8 | 9.1 | 6.8 |
|  | 2.0 | 47.7 | 37.6 | 24.1 | 24.2 | 12.2 | 15.3 | 5.7 | 9.2 | 1.6 | 5.2 | 9.6 | 8.5 |
|  | 3.0 | 46.8 | 36.6 | 24.0 | 23.9 | 12.3 | 15.3 | 6.0 | 9.4 | 2.0 | 5.4 | 10.0 | 9.4 |
|  | 4.0 | 46.3 | 36.0 | 23.9 | 23.6 | 12.4 | 15.3 | 6.1 | 9.5 | 2.1 | 5.6 | 10.2 | 10.0 |
|  | 5.0 | 46.0 | 35.6 | 23.8 | 23.4 | 12.4 | 15.3 | 6.2 | 9.6 | 2.3 | 5.7 | 10.4 | 10.4 |
|  | 6.0 | 45.7 | 35.2 | 23.7 | 23.3 | 12.4 | 15.2 | 6.2 | 9.6 | 2.4 | 5.8 | 10.5 | 10.8 |

For the validated simulations by Rauh et al ^17^, the drainage performance of the individual side hole rows was compared with the simulation results carried out in this study. However, due to different boundary conditions, which have a significant influence on the calculation, only a qualitative comparison is possible. Even if the numerical values for the positions for the Row IV and Tip entry openings are almost identical.

# Supplement B

## Name of side holes

An insertion length of 380.0 mm was selected for all cannula designs and a length of 70 mm for the connector with a 3/8" diameter (Figure 11C). The wire mesh within the cannula wall for flexible stiffening was not taken into consideration when setting up the simulation models.

In the first step, all relevant dimensions were taken from the real samples with round side holes (Figure 11A). To generate the respective 3D CAD models (Figure 2A-E), the value s=0.4mm was used for the wall thickness. The first row of four side holes (Row I) begins at a distance of ∆L_SH_=33mm or ∆L_SH_=25mm from the tip. The rows that follow continuously, i.e. with the same spacing (∆L_SH_), are incremented proximally to Row III or Row IV (Figure 11B). The side holes Q_i=1,4_ are always opposite each other in pairs.

## Geometry

After considering the drainage performance of the cannula with the circular side holes (Figure 2A) and their influence on the flow behaviour within the cannula, it was considered which parameters of this basic design could be varied in order to achieve an improvement. To ensure comparability with the standard cannula, the number and position of the rows and the number and position of the side holes have remained unchanged. The shape and/or size of the side holes has therefore been changed. Due to the limited surface area of the cannula, an increase in the diameter of the circle to only D_SH_=3mm was considered. Otherwise, the stability of the cannula would be impaired. The cross-sectional area was therefore only changed in the longitudinal direction L_SH_ so that either an ellipse or an elongated hole was created (Figure 2B,C). As a further variation, starting from an oblong hole, the area was reduced exponentially proximally on the one hand and distally on the other until a circular area was created again. (Figure 2D,E).

$$\sum_{i=2}^{4} A_{SH,i}=A_{SH,1}\cdot\left( 1+{1.2}^{i-1} \right)$$

(Eq. 2)

In the second step, the marketable ‘Lighthouse’ design (Figure 3A ), which has a total of six offset side holes in close proximity to the cannula tip, was also included in the investigation. The modelling was based on reverse engineering of the relevant dimensions of an original cannula. Furthermore, for the reference design ‘Multi-staged’ (Figure 2A) the effects on the flow were considered in the case that two or even three rows of side holes are closed, for example due to suction to the vessel wall. This situation was taken into consideration by modelling correspondingly fewer side holes in the basic geometry (Figure 3B,C).

Based on the 3D CAD models of the analysed variants, models of the fluid volumes were generated, which in turn, together with the physiological boundary conditions used, form the basis for the flow simulations. Corresponding planes were created in the flow volumes to analyse all relevant physical parameters of the converged simulations. This made it possible both to read out the numerical data and to display it graphically in contour plots.

## Simulation model

The structure of a simulation model, the individual physical boundary conditions and post-processing are described in more detail below. The non-Newtonian behaviour of the blood fluid requires a special mathematical model. Particular emphasis has been placed on its detailed derivation.

The position of the cannula within the flow volume (plenum) is visualised in Figure 12A. To ensure that only the pure drainage behaviour of the cannula can be investigated, the diameter of the plenum has been set to 25 times the outer diameter of the respective cannula and the length to a total of 950 mm. The symmetry axes of the plenum and the solid model of the cannula are congruent. ANSYS^®^ Fluent^®^ (ANSYS^®^ Inc) was used for the meshing with polyhedral mesh elements and the subsequent numerical flow simulation.

To rule out any influence of the volume network on the simulation results, the corresponding network parameters were determined for an operating point (3.0 L/min) as part of a network study to ensure this. The resulting network consists of 22.3∙10^6^ nodes. In addition to the independence with regard to the pressure loss, the two parameters y+ value: 1.3 and maximum aspect ratio: 89 confirm the required network quality.

## Boundary Conditions

The numerical calculation is based on the following physiological boundary conditions (BC). With regard to the physiological flow conditions, the cardiac output (CO) has been set at 5.0 l/min. It should be noted that the CO is split, with 1/3 allocated to the VCS and 2/3 to the VCI [32]. Variations in cardiac output, CVP or blood viscosity have no significant influence on the flow conditions within the cannula. This statement has been confirmed by a preliminary study using a CO of 2.5 l/min as an example. All surfaces of the simulation model must be assigned to the following boundary conditions (Figure 12B). At the 3/8" connector, the OUTLET type boundary condition for the ECMO flow of 1.0 to 6.0 L/min and additionally 2.5 L/min has been set for the drainage. With regard to the physiological flow situation in a femoral drainage, a boundary condition of type INLET with the corresponding velocity depending on the cardiac output (CO) has been selected. The velocity must be adapted to the diameter of the plenum by means of the cross-sectional area of the VC. The cross-sectional area is assumed to be circular and calculated with a diameter of d_VC_=30mm.

The factor 2/3 results from the fact that the CO is divided 1/3 into the VCS and 2/3 into the VCI ^32^. A boundary condition of type OPENING with a relative pressure of 0 Pa is defined at the VCS edge of the plenum. This ensures that any backflows that may form are taken into account. The reference pressure, i.e. the absolute vascular pressure, is made up of the ambient pressure (p_0_=101.325Pa) and the central venous pressure (CVP) (CVP=10mmHg). The no-slip boundary condition, i.e. the velocity at the wall surfaces is zero for the frictional flow, was selected for all walls.

Furthermore, a fluid "blood" has been defined that has a constant density ρ_Blood_=1.050kg/m³. Due to the special rheological properties of blood, its non-Newtonian behaviour should be taken into account. This is done by making the calculation of the dynamic viscosity μ dependent on the shear rate. The basis for the calculation is the Casson equation:

$$\mu\left( \dot{\gamma} \right)=\mu_{\infty}+\frac{\tau_{0}}{\dot{\gamma}}+2\cdot\sqrt[2]{\frac{\tau_{0}\cdot\mu_{\infty}}{\dot{\gamma}}}$$

(Eq. 3)

The variable $\mu_{\infty}$ is the blood viscosity at very high shear rates ($\mu_{\infty}$ → $\infty$). It is calculated according to ^33^, ^34^ as follows:

$$\mu_{\infty}=\frac{\mu_{pl}}{\left( 1-Hkt \right)^{1.969}}$$

(Eq. 4)

Hkt=0.45 was selected for the haematocrit (HKT) parameter for men in this study. The dynamic viscosity μ_pl_ of the blood plasma, which is also required, depends on the concentration c of the plasma proteins dissolved in the blood plasma, such as albumin, globulins and fibrinogen. With an assumed total protein concentration of human blood of c=7.3 g/100ml, $\mu_{pl}$ can be calculated using the following empirical formula according to ^33^:

$$\mu_{pl}=\mu_{H2O}\left( T \right)+0.14287\cdot c-0.016731\cdot c^{2}+0.0016\cdot c^{3}$$

(Eq. 5)

The dynamic viscosity of water can either be taken from standard tables such as the CRC Handbook of Chemistry and Physics for the temperature range of 20℃<T<100℃ or calculated according to ^35^:

$$log\left( \mu_{H2O} \right)=\frac{1,301}{998.333-8.1855\cdot\left( 20-T \right)+0.00585\cdot\left( 20-T \right)^{2}}-1.30233$$

(Eq. 6)

For the blood temperature T=37°C, the result is $\mu_{H2O}$=0.69cP. As the equation (Eq. 5) is a pure numerical value equation, only the numerical values have been used there. This results in $\mu_{pl}$=1.46cP and $\mu_{\infty}$=4.74cP. (Note: If the haematocrit value had been selected for women, $\mu_{\infty}$ would have been reduced by approx. 20 %).

The yield point t_0_ was determined as the last variable. Although it is independent of the temperature, it is strongly dependent on the fibrinogen concentration $c_{fib}$ and the haematocrit value. With the following empirical equation according to ^33^, ^34^ with an assumed $c_{fib}$ value of 0.27g/100ml, it is calculated as:

$$\tau_{0}=\frac{\left( Hkt-0.07 \right)^{2.779}}{10}\cdot\left( \frac{9.446\cdot c_{fib}}{1+5.161\cdot c_{fib}} \right)^{1.865}$$

(Eq. 7)

This means that all variables for the Casson equation (Eq. 3) are known. The value for the shear rate is determined within the numerical simulation and made available for the calculation of the dynamic viscosity $\mu$.

A steady state simulation in double precision with the standard two-equation shear stress transport (SST) turbulence model from Menter ^36^ was carried out for each volume flow.

## Post-Processing

The CFD-Post^®^ software from Ansys^®^ Inc was used in this study for the post-processing of all CFD simulations. MS-Excel^®^ (Microsoft^®^, Redmond, WA, USA) was used to process all the data and prepare it graphically.

For the evaluation of the mass flow of the individual rows of side holes, corresponding evaluation planes perpendicular to the flow within the cannula were defined. They are visualised in Figure 11B as an example for the Maquet^®^ cannula (19 Fr.) (red dotted line). Their position is centred between the rows of side holes and the tip of the cannula. The most proximal plane lies so far behind the corresponding row of side holes that a fully developed flow is ensured there. By simply subtracting the previous mass flow from the current mass flow, the actual mass flow through the four side holes of the row currently under consideration has been calculated. Due to the symmetrical design and positioning of the rows of side holes, symmetrical inflow behaviour can also be assumed.

# Supplement C

## Stasis treshold

In this study, as in Markl et al ^29^ , a threshold value of 0.1 m/s has been chosen as the value below which stasis occurs. In addition, the effect of doubling or halving this threshold value on the size of the stasis-prone zone has been investigated. The following figures show the distribution of velocity within the cannula for different stasis threshold values for the cross-sectional area of the respective cannula variant.

For the combination of 19 Fr. and 5.0 L/min ECMO flow (Figure 13A), there are clear differences in the cannula designs. The reference cannula (MSC-LH-CI) has an almost stasis-free velocity distribution for the doubled stasis limit, which extends to the tip of the cannula. Thrombi could form only in ROW I. With the two variants MSC-LH-EL and MSC-LH-SL, the stasis-safe zone does not even extend completely to the most proximal side hole row (ROW III). This means that a very large area up to the tip is at risk of clotting. With the two variants MSC-LH-SP and MSC-LH-SD, on the other hand, the safe area extends to the side hole row ROW II. The difference between them is considered to be minor and not decisive.

For the selected stasis threshold value, the reference design (MSC-LH-CI) improves even further by not reaching it before the cannula tip. Due to the smaller area of the elliptical side holes compared to the sloth holes, the stasis-safe zone increases to the beginning of the second row of side holes. However, the area up to the tip, including ROW II, remains at risk of clotting. In the MSC-LH-SL variant, the risk zone changes negligibly. In the MSC-LH-SP and MSC-LH-SD variants, the influence of the area of the side holes is very clear. In the MSC-LH-SD variant, the increase in area at ROW I reduces flow resistance, allowing a higher velocity to develop and thus enlarging the stasis-safe area.

If the selected threshold value is halved, there is hardly any change in the MSC-LH-CI variant. In the MSC-LH-EL variant, the area at risk of stasis is reduced only insignificantly. The MSC-LH-SL variant improves to a similar level as the MSC-LH-EL variant. The MSC-LH-SP and MSC-LH-SD variants also show a slight improvement in terms of the extent of the clotting zone.

For the combination of 23 Fr. and 1.0 L/min ECMO flow (Figure 13B), the areas at risk of stasis increase significantly. Even with the reference cannula (MSC-LH-CI), a zone at risk of clotting now forms from the side hole row ROW III to the tip of the cannula. Depending on the stasis threshold, the zone does decrease. However, a significant zone with velocities below 0.05 m/s remains between ROW I and the tip.

It is noticeable that the MSC-LH-EL, MSC-LH-SL and MSC-LH-SP variants have an almost identical area from ROW IV to the cannula tip in which clotting is a risk, regardless of the threshold value. The MSC-LH-SD variant, on the other hand, can reduce this area at risk of stasis by one row of side holes.

## Low flow zone

In addition to the solely qualitative description (Figures 13A, B) of the zones with low or no flow velocity, the extent of these zones is also shown quantitatively in Figures 14A, B, C. For this purpose, the core velocity, i.e. the velocity along the centre line of the cannula, was read out and plotted as a diagram with normalised axes ($v_{max}=5.0 \frac{m}{s} ;L=350 mm$). The stasis threshold is visualised as a dotted line.

Comparing the lengths from the tip in the proximal direction to the position where the terminal velocity for stasis first occurs for the combination of 19 Fr and 5.0 L/min ECMO flow (Figure 14A,B), it can be seen that, with the exception of the reference design (MSC-LH-CI), all other design variants reach the low flow zone after just 35 mm. For the MSC-LH-SL variant, the stasis-prone zone begins just 94.5 mm from the tip. For the MSC-LH-EL (70.0 mm) and MSC-LH-SP (66.5 mm) variants, the distance is shorter and thus the stasis-prone zone is smaller. Of the new variants, MSC-LH-SD has the shortest distance at 35.0 mm.

For the combination of 23 Fr. and 1.0 L/min ECMO flow, the lengths increase significantly. Even for the reference design, there is a distance of 28 mm between the start of the low flow zone and the tip. The lengths of the stasis-critical zones for the MSC-LH-EL and MSC-LH-SP variants (98.0 mm) and the MSC-LH-SL variant (101.5 mm) are approximately 350% greater than those of the MSC-LH-CI variant.

## Pressure drop

For the volume flow rate of *Q*=5.0 L/min used in this study, the pressure loss between the cannula connection and the first proximal row of side holes (Row III) is due to friction in the pipe flow. For improved comparability, in figure 15 the values for pressure and length have been normalised to their maximum values (p=200 mmHg, L=350 mm). For the side hole variants MSC-LH-EL, MSC-LH-SL and MSC-LH-SP, drainage occurs almost exclusively in the first proximal row of side holes. The proportion of the distal side hole rows in the aspirated volume is marginal. This behaviour is also accompanied by a significant pressure drop. With the MSC-LH-SD side hole variant, a large proportion of blood is still drained through the proximal side hole row. However, due to the increase in the proximal area, a significant volume is also aspirated through the side hole row Row III. This behaviour is due to a significant pressure reduction, still possible in Row III.

Figure 10

Title:	Pressure loss curve

Legend:	(A) Compared to the reference design (MSC-LH-CI), all design variants show better behaviour in terms of pressure loss. Even at a flow rate of 6 L/min, it is above the critical value of 100 mmHg. Furthermore, the direct dependence of the pressure loss on the design can be recognised. (B) With a reference cannula (MSC-LH-CI) suctioned onto the vessel wall, the pressure loss is significantly worse. However, it is slightly better than for a free-suction cannula with the lighthouse design.

Figure 11

Title:	Reverse-engineering of a standard drainage cannula

Legend:	(A) A marketable 23 Fr. drainage cannula from Maquet^®^ was used to generate a CAD model using reverse engineering. The diameters and positions of the side holes were measured. The number of rows of side holes and the spacing depend on the respective outer diameter of the cannula are identical for all design variants.

(B) Starting from the tip of the cannula, the rows are incremented proximally. To determine the mass flows of the individual rows of side holes, vertical planes (red dotted line) have been defined between them to calculate the respective volume flows as a quotient of the mass flow and the density.

(C) All design variants have an insertion length of 380 mm. This is followed by the 70 mm long connector with its 3/8" connection.

Figure 12

Title:	Visualisation of fluid domain and boundary conditions

Legend:	(A) The flow volume (plenum) consists of a cylindrical body with a total length of 950 mm. The diameter is 25 times the outer diameter of the respective channel. This ensures that the drainage behaviour can be investigated without being distorted by effects close to the wall. (B) The respective boundary conditions are assigned to the individual surfaces of the flow volume.

Figure 13

Title:	Velocity distribution for different stasis critical tresholds

Legend:	The distribution of velocity within each cannula variant is used to visualise the change in size of the stasis-prone zone when the threshold value for stasis is doubled or halved. (A) Combination of 19 Fr. and 5.0 L/min ECMO flow, (B) Combination of 23 Fr. and 1.0 L/min ECMO flow

Figure 14

Title:	Core velocity within cannula

Legend:	To quantitatively describe the extent of the zones at risk of stasis, the core velocity, i.e. the velocity along the centre line of the cannula, is read out and plotted as a diagram with normalised axes ($v_{max}=5.0 \frac{m}{s} ;L=350 mm$). The stasis threshold is visualised as a dotted line.

(A) Combination of 19 Fr. and 5.0 L/min ECMO flow, (B) Combination of 23 Fr. and 1.0 L/min ECMO flow

Figure 15

Title:	Pressure reduction due to the side holes

Legend:	The visualisation of the pressure curve along the axis of rotation (centre line) for a flow rate of 5.0 L/min (A) shows the greatest pressure drop for the proximal side hole row (Row III) for all design variants. Only in the design with proximal surface reduction (MSC-LH-SD -19Fr-V) is a significant pressure drop also visible in the following row (Row II). Only then does the pressure drop to the level in the plenum, causing the flow to come to a standstill.

Even when the diameter is increased to 23 Fr. with the same flow rate of 5.0 L/min (B), the proximal side hole row (Row IV) shows the greatest pressure reduction along the centre line and thus the reduction in flow velocity for all design variants.
